# Elevated White Blood Cell Counts Despite Normal C-Reactive Protein Independently Predict Metabolic Syndrome and Myocardial Infarction: Evidence for Genetic Susceptibility and Dietary Modification in a Prospective Cohort

**DOI:** 10.3390/nu18183040

**Published:** 2026-09-17

**Authors:** In Seok Bang, Sunmin Park

**Affiliations:** 1 Research Institute for Basic Sciences, Hoseo University, Asan 31499, Republic of Korea; 2 Department of Food and Nutrition, Obesity/Diabetes Research Center, Hoseo University, Asan 31499, Republic of Korea

**Keywords:** white blood cells, C-reactive protein, immune dysregulation, gene-diet interaction, genetic susceptibility, precision nutrition

## Abstract

**Background/Objectives:** Elevated white blood cell (WBC) counts and C-reactive protein (CRP) are established inflammatory markers associated with cardiometabolic disease. However, whether elevated WBC remains prognostically important without CRP elevation is unclear. This study aimed to examine whether elevated WBC despite normal CRP would predict incident metabolic syndrome (MetS) and myocardial infarction (MI), and to investigate its genetic basis and dietary modifiability. **Methods**: This prospective cohort study included 54,744 Korean adults with a mean follow-up of five years. Participants were classified as WBC-Only Elevated (WOE; WBC ≥ 6.2 × 10^9^/L and CRP < 1.0 mg/dL) or normal WBC and CRP (Low Risk) at baseline. Cox proportional hazards models estimated risks of incident MetS and MI. Genome-wide association studies (GWAS) identified genetic variants associated with WOE, from which weighted genetic risk scores (GRS) were constructed and replicated in the UK Biobank (n = 352,394). GRS–diet interactions were evaluated using a validated 106-item semi-quantitative food frequency questionnaire and 24 h dietary recalls. **Results**: WOE was independently associated with increased risks of MetS (HR = 1.82, 95% CI: 1.57–2.11) and MI (HR = 1.60, 95% CI: 1.25–2.04; both *p* < 0.001) compared with Low Risk. Immune-related variants at 17q21.1 and 6p21.3 were replicated in the UK Biobank. Among individuals with high genetic risk, adherence to a Korean-style balanced diet and higher overall diet quality attenuated genetic susceptibility to WOE by 16% (*p* = 0.042) and 30% (*p* = 0.031), respectively. **Conclusions:** Elevated WBC despite normal CRP independently predicted incident MetS and MI. These findings suggest that incorporating WBC alongside CRP in inflammatory risk assessment, together with genetic and dietary information, may support more targeted cardiometabolic risk stratification.

## 1. Introduction

Chronic low-grade inflammation is increasingly recognized as a central mechanism linking metabolic dysregulation to cardiovascular risk. Previous studies have demonstrated that inflammatory pathways, including NF-κB activation, pro-inflammatory cytokine release, and endothelial dysfunction, promote insulin resistance and atherosclerotic progression independently of traditional risk factors [1,2]. C-reactive protein (CRP) is the most widely used clinical biomarker of systemic inflammation. Multiple large prospective studies have demonstrated associations between elevated CRP and increased risks of MetS, type 2 diabetes, and CVD [3,4]. However, CRP primarily reflects the hepatic acute-phase response driven by IL-6 and other inflammatory cytokines [5] and may rise transiently in response to acute infection, tissue injury, and obesity, independently of sustained immune activation. Furthermore, CRP levels show substantial ethnic variation; East Asian populations exhibit characteristically lower baseline CRP concentrations than Western populations [6], potentially limiting the sensitivity of CRP-based risk stratification in Korean and other Asian cohorts and leading to systematic underestimation of cardiometabolic risk in this population [3].

Circulating white blood cell (WBC) count is a readily available measure of systemic leukocyte mobilization and innate immune activation. A meta-analysis of 168,000 patients demonstrated significant associations between elevated WBC parameters and MetS [7], and prospective studies have reported that elevated WBC independently predicts incident type 2 diabetes and cardiovascular events [8,9]. Importantly, these associations persist even within the conventional normal reference range, suggesting that WBC captures a dimension of immune activation not fully reflected by CRP. In our previous longitudinal study using the same Korean cohort, we identified a WBC threshold of 6.2 × 10^9^/L above which the risk of incident MetS increased significantly [10], providing the biological rationale for the present phenotype definition. Because WBC and CRP reflect complementary but distinct components of the inflammatory response [11], individuals with elevated WBC despite normal CRP may represent a subgroup with persistent leukocyte-predominant immune activation that is not detected by conventional CRP-based screening.

Despite growing evidence that both WBC and CRP independently predict cardiometabolic outcomes [12,13], the prognostic significance of discordant WBC–CRP profiles (specifically, elevated WBC in the absence of CRP elevation) remains poorly characterized. To our knowledge, no large prospective study has systematically evaluated whether this discordant immune profile, hereafter termed WBC-Only Elevated (WOE), constitutes a clinically meaningful and independent risk state for incident MetS and MI. Furthermore, the genetic architecture underlying susceptibility to this phenotype has not been investigated, and whether dietary patterns modify genetic risk within this immune phenotype remains unknown.

Immune activation phenotypes are further influenced by genetic susceptibility [14] and dietary exposures [15]. Accumulating evidence suggests that gene–diet interactions modify inflammatory responses and cardiometabolic risk, providing a rationale for examining whether dietary patterns attenuate genetic susceptibility to WOE and its associated cardiometabolic risk [16,17]. The aim of this study was therefore to (1) prospectively evaluate whether the WOE phenotype predicts incident MetS and MI in a large Korean cohort; (2) identify genetic variants underlying WOE susceptibility using GWAS with UK Biobank replication; and (3) examine whether dietary patterns modify genetic susceptibility to WOE using weighted genetic risk scores (GRS) in gene–diet interaction analyses.

## 2. Materials and Methods

### 2.1. Study Population, Design, and Sample Size

This prospective cohort study utilized data from the Health Examinees Study (HEXA). Study procedures were approved by the Institutional Review Boards of the Korean National Research Institute of Health for HEXA (KBP-2015-055), the UK Biobank (UKBB) Ethics Committee (11/NW/0382), and the Hoseo University Ethics Committee (1041231-150811-HR-034-01 for HEXA; 1041231-240820-BR-183-01 for UKBB), in accordance with the Declaration of Helsinki. All participants provided written informed consent.

Baseline measurements were established at the 1st recruitment (2004–2013), with follow-up at the 2nd recruitment (2012–2016; Figure S1). The inclusion criteria were (1) aged 40–75 years at baseline; (2) complete WBC and CRP measurements at both recruitment visits; and (3) available follow-up data for MetS and MI outcomes. The exclusion criteria were applied sequentially across two parallel data streams. In the 1st recruitment stream, 107,584 participants without 2nd recruitment data and 10,660 with incomplete WBC or CRP measurements were excluded from the initial pool of 173,195, leaving 54,951 participants. A further 85 participants lacking WBC measurements at follow-up were excluded, yielding 54,866 participants with complete inflammatory marker data at both time points. Of these, 122 participants without matching 2nd recruitment records were additionally excluded, resulting in a final analytic sample of 54,744 individuals. In the 2nd recruitment genetic stream, 58,701 participants had genetic measurements available. After excluding 3845 participants without matching records and 112 with incomplete genetic variants, 54,744 participants were confirmed for genetic analyses, consistent with the inflammatory marker sample. Participants with prevalent MetS or MI at baseline were excluded from respective incident disease analyses. A sensitivity analysis further excluded 1934 participants with cancer diagnoses, yielding a sensitivity sample of 52,810, as malignancy and its treatments can alter immune cell counts through mechanisms distinct from metabolic disease (Figure S1).

Sample size calculations (G*Power v. 3.1.9.2; two-sided α = 0.05, power = 0.80, HR = 1.20, event rates of 15% for MetS and 3% for MI) indicated a minimum of 8500 participants, confirming adequate statistical power for primary analyses, subgroup stratification, and covariate adjustment [18].

### 2.2. Definition of Immune Phenotypes

Immune phenotypes were defined using previously established population-specific thresholds for WBC (6.2 × 10^9^/L) and CRP (1.0 mg/dL). The WBC threshold was identified in our previous longitudinal KoGES study as the level above which the risk of incident metabolic syndrome increased significantly and was subsequently validated in the independent KoGES Ansan/Ansung cohort [19,20]. The CRP threshold was selected based on established reference ranges for low-grade inflammation in Korean adults [14,15,16]. Participants were classified into four groups: Low Risk (WBC < 6.2 × 10^9^/L and CRP < 1.0 mg/dL; n = 21,828), WBC-Only Elevated (WOE; WBC ≥ 6.2 × 10^9^/L and CRP < 1.0 mg/dL; n = 32,187), Acute Inflammation (WBC < 6.2 × 10^9^/L and CRP ≥ 1.0 mg/dL; n = 154), and Both Elevated (WBC ≥ 6.2 × 10^9^/L and CRP ≥ 1.0 mg/dL; n = 575). The Acute Inflammation group (n = 154) was excluded from prospective analyses due to its small sample size. Furthermore, elevated CRP in the absence of leukocytosis more likely reflects transient acute inflammatory responses rather than the chronic subclinical immune activation investigated in this study. For replication analyses in the UK Biobank, population-specific thresholds (WBC ≥ 7.0 × 10^9^/L and CRP ≥ 3.0 mg/L) were applied to account for differences in hematological and inflammatory marker distribution between East Asian and European populations, consistent with previous studies [21,22,23]. This yielded Low Risk (n = 222,104) and WOE (n = 130,290) groups.

### 2.3. MetS and MI Definitions

MetS was defined according to the revised National Cholesterol Education Program Adult Treatment Panel III (NCEP-ATP III) criteria, requiring the presence of three or more of the following five components: (1) abdominal obesity, defined as waist circumference ≥ 90 cm in men or ≥85 cm in women using Asian-specific cutoffs; (2) elevated triglycerides ≥ 150 mg/dL or use of lipid-lowering medication; (3) reduced HDL-cholesterol < 40 mg/dL in men or <50 mg/dL in women; (4) elevated blood pressure ≥ 130/85 mmHg or use of antihypertensive medication; and (5) elevated fasting glucose ≥ 100 mg/dL or use of antidiabetic medication [24]. MI was ascertained by self-reported physician diagnosis. Incident cases were participants who had no MetS or MI at baseline who developed these conditions by follow-up.

### 2.4. Clinical Measurements

Standardized questionnaires administered by trained personnel captured demographic information (age, sex, residential area), socioeconomic status (education level, monthly household income, occupation), and lifestyle behaviors including smoking status (never, former, current), alcohol consumption (g/day), and physical activity [10]. Body weight, height, and waist circumference were measured by trained staff following standardized protocols, with participants in light clothing and without shoes [25]. Following ≥12 h overnight fasting, venous blood samples were collected and analyzed using a Hitachi 7600 Automatic Analyzer (Hitachi Ltd., Tokyo, Japan) [25]. Measurements included lipid profiles (LDL-C, HDL-C, triglycerides), fasting glucose, liver enzymes (GOT, GPT, γ-GTP), renal markers (BUN, creatinine), and HbA1c. WBC counts were measured using heparin-treated blood samples on an Automated Hematology Analyzer. Serum high-sensitivity CRP was quantified using enzyme-linked immunosorbent assay (ELISA).

### 2.5. Dietary Assessment

Dietary intake was assessed using a validated 106-item semi-quantitative food frequency questionnaire (SQFFQ) specifically developed and validated for the Korean population [26]. The SQFFQ demonstrated acceptable reproducibility, with median correlation coefficients of 0.45 between two administrations one year apart, and acceptable validity against 12-day diet records, with de-attenuated correlation coefficients ranging from 0.23 to 0.64 across nutrients (median r = 0.39) and less than 7% of participants misclassified into distant quartiles for most nutrients [27]. Trained technicians administered the SQFFQ through structured interviews capturing usual dietary intake over the preceding 12 months, with consumption frequency and portion sizes recorded using pictorial references. Separately, 24 h dietary recalls were conducted by skilled technicians to assess recent nutrient intake. Daily energy and nutrient intakes were calculated from 24 h recall data using the Computer-Aided Nutritional Analysis Program (CAN-Pro 3.0; Korean Nutrition Society) based on the Korean food composition database [20]. Energy intake was expressed as a percentage of the estimated energy requirement (EER), calculated using age- and sex-level-specific equations from the 2015 Dietary Reference Intakes for Koreans [20]. Macronutrient intakes were expressed as percentages of total energy intake (En%), and micronutrient intakes were expressed as absolute daily amounts.

For dietary pattern analysis, the 106 food items from the SQFFQ were aggregated into 29 food groups based on nutritional similarity and culinary use in Korean cuisine. Principal component factor analysis with Varimax orthogonal rotation was applied to the 29 food groups (eigenvalue > 1.5; factor loading ≥ 0.40), yielding four dietary patterns [26]. Korean-style balanced diet (KBD), characterized by high intake of fermented soybeans, fish, seafood, meat, seaweed, vegetables, kimchi, and mushrooms; Western-style diet (WSD), featuring noodles, soups, meats, processed meats, and fast foods; egg-and-dairy-rich diet (EDR), with elevated consumption of eggs, milk, beverages, fruits, and nuts; and rice-main diet (RMD), consisting predominantly of refined rice [28,29].

Diet quality was assessed using the Korean-adapted Healthy Eating Index (HEI; range 0–150), developed and validated for Korean dietary assessment [30,31]. The HEI comprises three domains scored against age- and sex-specific criteria derived from the Korean Dietary Guidelines and the 2015 Dietary Reference Intakes for Koreans [31]: (1) the adequacy domain evaluates intake of recommended food groups, including breakfast frequency, mixed grains, fruits, vegetables, kimchi, seaweed, fish, meat and eggs, beans, milk products, and nuts, scored using food group serving sizes derived from the SQFFQ; (2) the moderation domain assesses intake of components to limit, including saturated fatty acids, polyunsaturated fatty acids, sodium, energy from sweets and beverages, noodle intake, and alcohol, scored using nutrient and energy intake data from 24 h dietary recalls; and (3) the balance domain evaluates overall dietary balance, including energy intake relative to estimated energy requirements, and vitamin C, dietary fiber, calcium, carbohydrate, and fat as percentages of energy intake, scored using nutrient data from 24 h dietary recalls. Total scores range from 0 to 150, with higher scores indicating better overall diet quality [30,32]. The Dietary Inflammatory Index (DII) was computed from 38 food and nutrient components using published inflammatory weights [33].

### 2.6. Genetic Analysis

Genomic DNA was genotyped using the Korea Biobank Array (KoreanChip; Affymetrix, Santa Clara, CA, USA) [34]. Only imputed variants with INFO > 0.8 were retained. Population stratification was addressed using the first 10 principal components as covariates. Logistic GWAS compared WOE (n = 32,187) versus Low Risk (n = 21,828) under an additive genetic model, adjusting for age, sex, BMI, lifestyle factors, and principal components (genome-wide significance threshold: *p* < 5 × 10^−8^). Independent signals were identified by LD pruning (r^2^ < 0.01). Weighted genetic risk scores (GRS) were constructed separately for missense and 3′-UTR variant categories using effect-size-weighted risk allele sums, and participants were categorized into GRS tertiles. UKBB replication used logistic regression with comparable covariate adjustment; complementary linear regression examined associations between variants and circulating WBC counts in both cohorts.

### 2.7. Statistical Analysis

All analyses were performed using SAS v9.3. Participants with missing data for WBC, CRP, MetS, or MI were excluded; missing lifestyle variables were imputed using means (continuous) or modes (categorical). Three hierarchical covariate adjustment models were applied sequentially: Model 1 (age, sex, residence area, follow-up period); Model 2 (plus education, income, BMI); Model 3 (plus daily energy intake, regular exercise, smoking status, alcohol consumption). Baseline characteristics were compared across immune phenotype groups using chi-squared tests for categorical variables and ANCOVA with Tukey’s post hoc tests for continuous variables. Cross-sectional associations between immune phenotypes and prevalent MetS or MI were examined using logistic regression with Low Risk as reference, reporting odds ratios (ORs) and 95% confidence intervals (CIs) under Models 2 and 3.

For prospective analyses, Cox proportional hazards regression estimated hazard ratios (HRs) and 95% CIs for incident MetS and MI occurring between the 1st and 2nd recruitments, using the Low-Risk group as the reference and applying Models 1–3 sequentially. The Acute Inflammation phenotype (n = 154) was excluded from prospective analyses due to small sample size and its representation of acute rather than chronic immune activation. Proportional hazards assumptions were verified using Schoenfeld residuals. Cumulative incidence across immune phenotypes was visualized using Kaplan–Meier curves with log-rank tests.

Dietary modification of immune phenotype–disease associations was examined by testing interactions between WBC/CRP phenotypes and dietary patterns using likelihood ratio tests comparing Cox models with and without multiplicative interaction terms. Twelve interactions were evaluated: four dietary patterns (KBD, WSD, PBD, RMD) × three immune phenotypes (Low Risk, WOE, Both Elevated) for each outcome (MetS and MI). Significant interactions (*p* < 0.0042 after Bonferroni correction for 12 tests) prompted stratified Cox regression analyses calculating HRs separately within low- and high-intake categories of each dietary pattern under fully adjusted Model 3.

Gene–diet and gene–lifestyle interactions were assessed using multiplicative interaction terms (GRS × dietary pattern; GRS × lifestyle factor) in logistic regression models, with likelihood ratio tests and a Bonferroni correction (*p* < 0.00625 for 8 tests: 4 dietary patterns + 4 lifestyle factors). If interactions were significant, stratified analyses were conducted within GRS tertiles.

## 3. Results

### 3.1. Baseline Characteristics of the Participants by Inflammatory Phenotype

Four immune phenotypes were identified: Low Risk (n = 21,828), WOE (n = 32,187), Both Elevated (n = 575), and Acute Inflammation (n = 154). Compared to Low Risk, the WOE group had higher proportions of men (37.1% vs. 28.9%) and less favorable metabolic profiles, including higher BMI (23.6 vs. 24.1 kg/m^2^), waist circumferences (79.5 vs. 81.6 cm), SBP (122 vs. 123 mmHg), and DBP (75.0 vs. 76.6 mmHg), and serum TG (116 vs. 129 mg/dL), HDL (55.0 vs. 52.7 mmHg), AST (23.0 vs. 24.1 mg/dL), ALT (20.8 vs. 23.4 mg/dL), γ-GTP (32.7 vs. 31.0 U/L), and homocysteine (8.53 vs. 9.36 μM; *p* < 0.001; Table 1). The Both Elevated group demonstrated the most adverse cardiometabolic profile, with the highest prevalence of MetS (20.8% vs. 12.3%) and MI (3.4% vs. 2.7%) compared to Low Risk (Table 1).

### 3.2. Dietary Intake, HEI, and Lifestyle by Inflammatory Phenotypes

Total energy intake did not differ significantly between WOE and Low-Risk groups (99.1% vs. 99.0% of EER; Table 2). However, WOE participants showed higher crude intakes of several micronutrients compared to Low Risk, including flavonoids (45.1 vs. 43.4 mg/day), vitamin C (110 vs. 106 mg/day), vitamin E (8.28 vs. 8.03 mg/day), vitamin D (8.95 vs. 8.62 µg/day), fiber (15.6 vs. 14.3 g/day), and calcium (487 vs. 477 mg/day; all *p* < 0.001; Table 2). These higher absolute intakes likely reflect greater overall food consumption associated with higher BMI in the WOE group. After adjustment for energy intake, age, sex, and BMI, the WOE group showed significantly lower intakes of these micronutrients than Low Risk (Table S1), suggesting poorer dietary quality relative to energy needs despite higher absolute intake. The WOE group also showed higher sodium intake (2.53 vs. 2.39 g/day) and glycemic index (49.4 vs. 48.3; both *p* < 0.001) compared to the Low-Risk group.

Regarding dietary patterns, WOE participants showed higher adherence to WSD (37.9% vs. 30.8%) and lower adherence to PBD (29.2% vs. 33.3%; both *p* < 0.001) compared to Low Risk (Table 2). Overall HEI scores were modestly lower in the WOE group than the Low-Risk group (90.8 vs. 91.9), driven by lower adequacy (35.0 vs. 35.4) and moderation scores (42.4 vs. 43.1; both *p* < 0.001). WOE participants showed lower physical activity (15.5 vs. 18.3 min/day), higher alcohol intake (14.6 vs. 13.1 g/day), and higher current smoking prevalence (12.9% vs. 6.3%; all *p* < 0.001) compared to Low Risk (Table 2).

### 3.3. Prospective Associations Between WOE and Clinical Outcomes (MetS or MI)

**Figure 1:**
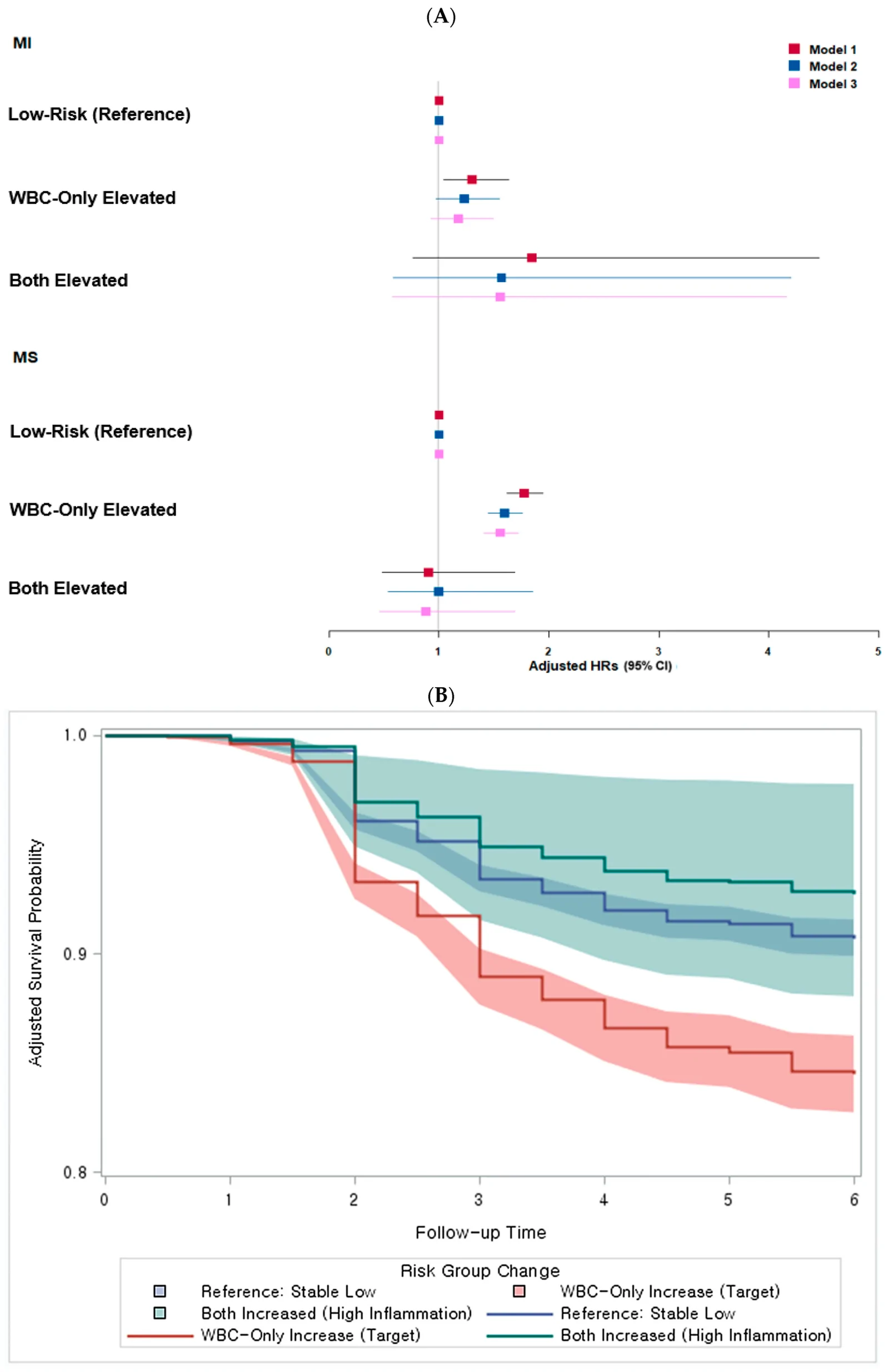

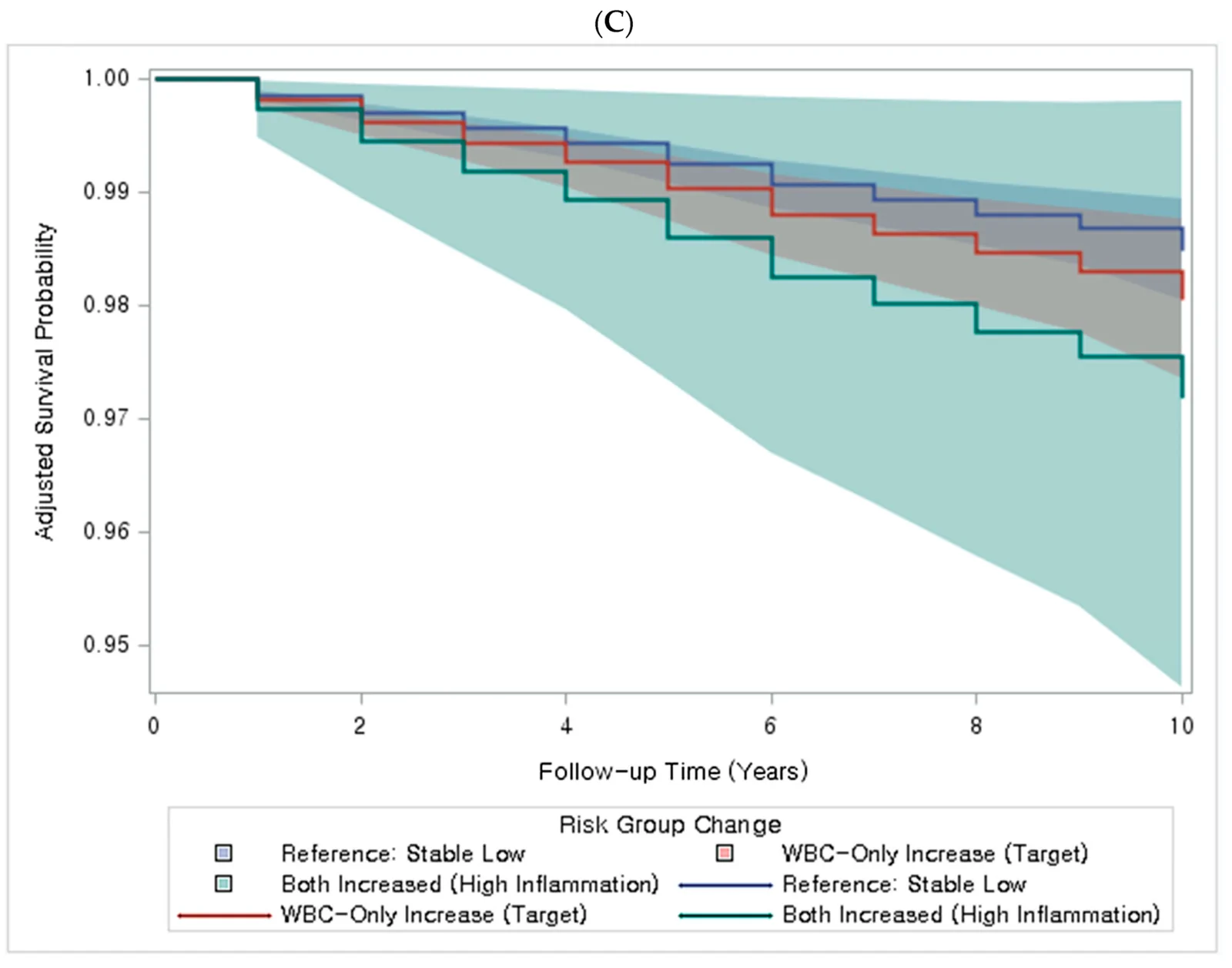
Associations of immune phenotypes by white blood cell (WBC) and serum C-reactive protein (CRP) levels with metabolic syndrome (MetS) and myocardial infarction (MI). (**A**) Forest plot of adjusted hazard ratios (HRs) and 95% confidence intervals (CIs) for immune phenotypes. (**B**) Kaplan–Meier survival curves for incident MetS. This plot illustrates the adjusted survival probability for developing MetS over a 5-year follow-up period across different WOE groups. (**C**) Kaplan–Meier survival curves for incident MI. This plot illustrates the adjusted survival probability for developing MI over a 5-year follow-up period across different WOE groups.

WOE independently predicted incident MetS (HR = 1.82, 95% CI: 1.57–2.11) and MI (HR = 1.60, 95% CI: 1.25–2.04) compared to the Low-Risk group in fully adjusted models (both *p* < 0.001; Figure 1A). Kaplan–Meier curves demonstrated progressive divergence in cumulative incidence across phenotypes over follow-up (log-rank *p* < 0.001; Figure 1B,C). Sensitivity analyses excluding cancer patients yielded consistent results (Figure S2A–C).

Low-Risk (normal-WBC/normal-CRP) group as the reference, WBC-Only Elevated (WOE; high-WBC/normal-CRP), and Both Elevated (high-WBC/high-CRP) groups. Model 1 adjusts for demographics, Model 2 for covariates in Model 1 plus metabolic factors, and Model 3 for covariates in Model 2 plus lifestyle factors.

### 3.4. Genetic Variants Associated with WOE

GWAS identified multiple loci associated with WOE at *p* < 5 × 10^−8^, with no genomic inflation (λ = 1.022; Figure S3A,B). The strongest associations were observed at 17q21.1 (*GSDMB–IKZF3* region; *p* = 3.39 × 10^−22^), 6p21.3 (*MHC* region; *p* = 2.05 × 10^−10^), and 2q21.3 (*MCM6–LCT* region; *p* = 1.73 × 10^−13^; Table 3 and Table S2). These loci predominantly map to immune regulatory regions.

GWAS analysis identified 13 genome-wide significant variants (Table 3), including three intronic variants that were excluded from GRS construction due to uncertain functional consequences. The remaining ten variants with predicted functional relevance were selected: five missense variants (*GCKR* rs1260326, *LCT* rs3754689, *HLA-C* rs1050428, *HLA-DQA1* rs10093, *GSDMB* rs56030650) and five 3′-UTR variants (*UBXN4* rs76260229, *DARS1* rs309133, *PSMD3* rs7021, *CSF3* rs25645, *MED24* rs2302777), as these variant classes directly alter protein function or post-transcriptional gene regulation (Table 3; Figure S3C). UK Biobank replication confirmed consistent risk allele directions, although two variants (rs76260229, rs309133) did not reach statistical significance (Table 3). Linear GWAS of WBC counts showed concordant associations in both cohorts (Table S3).

**Figure 2:**
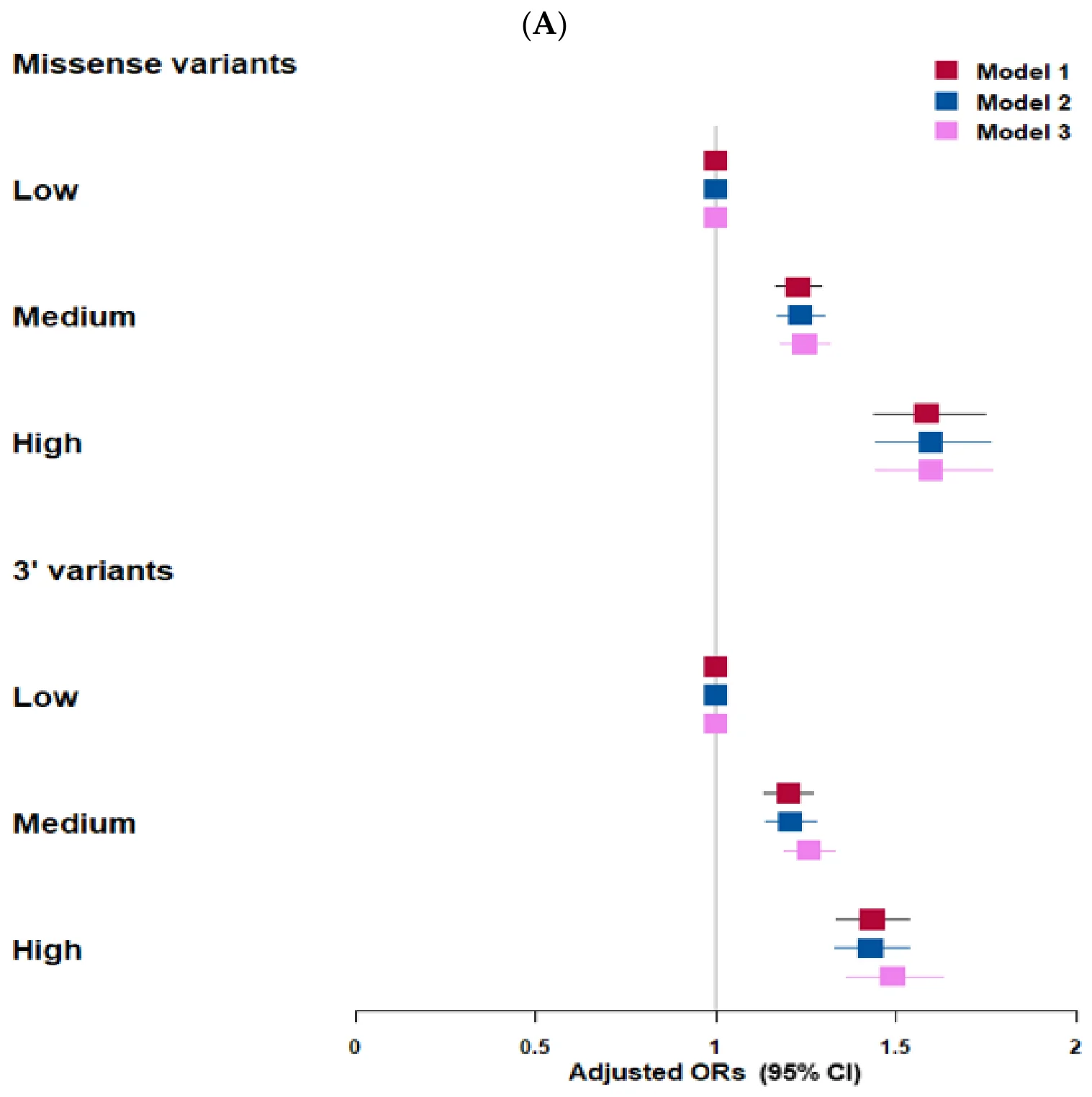

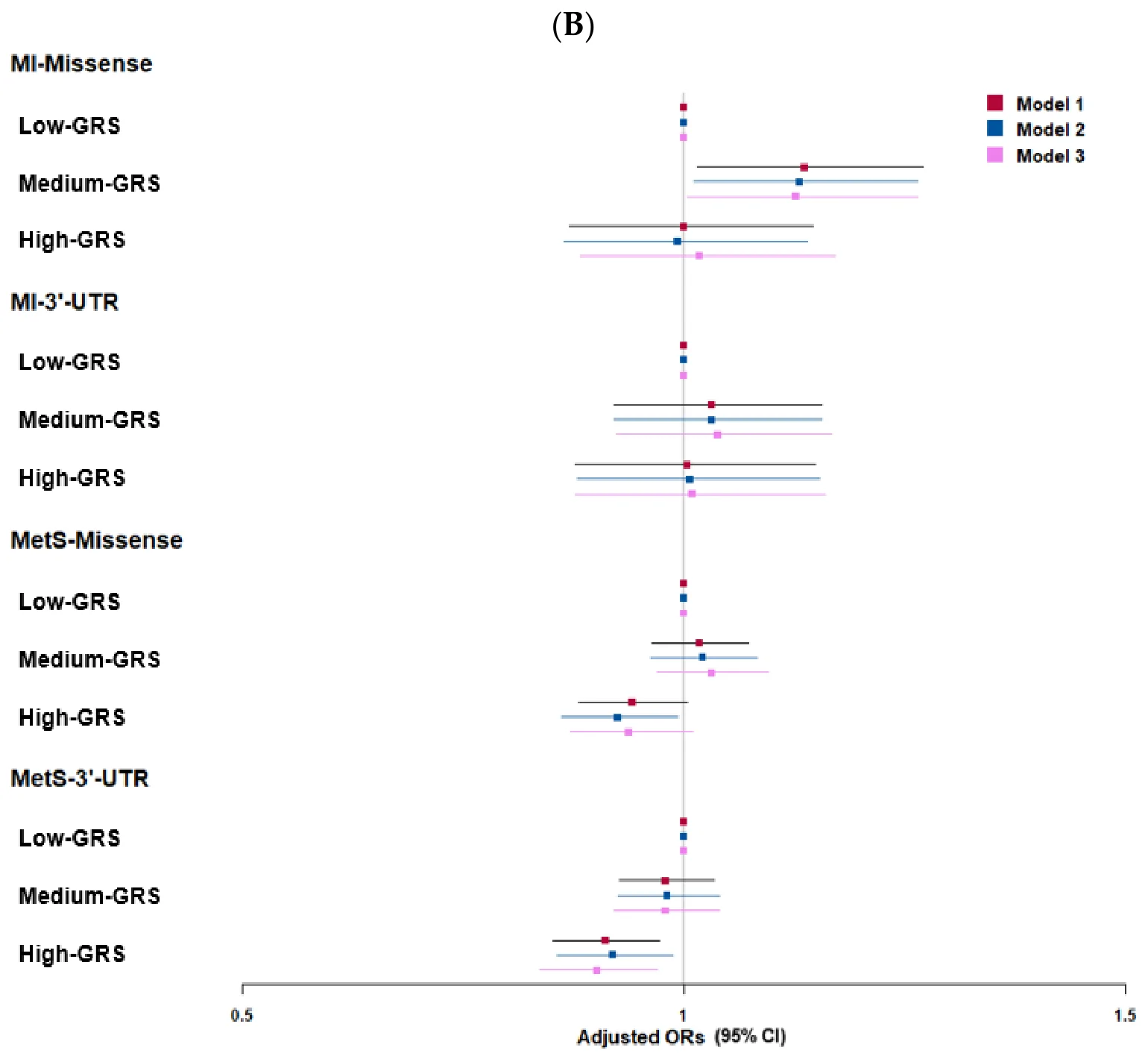
Association of weighted genetic risk scores (GRS) with immune activation phenotypes, metabolic syndrome (MetS), and myocardial infarction (MI). (**A**) Adjusted odds ratio (OR) and 95% confidence intervals (CI) of GRS with missense variants and 3′-UTR variants for WBC-Only Elevated (WOE; high-WBC/normal-CRP). (**B**) Adjusted ORs and 95% CI of the GRS with Missense and 3′-UTR variants for MetS and MI. It examines the relationship between the GRS derived from the identified loci and the WOE phenotype, as well as the cardiometabolic outcomes. All associations are adjusted using Model 3 (the fully adjusted model, accounting for demographic, metabolic, and lifestyle factors) to assess the independent effect of genetic risk on cardiometabolic outcomes.

Both missense and 3′-UTR GRS showed graded associations with WOE prevalence across tertiles: high missense GRS (OR = 1.47, 95% CI: 1.37–1.58) and high 3′-UTR GRS (OR = 1.50, 95% CI: 1.37–1.64), both *p* < 0.001 (Figure 2A). High missense and 3′-UTR GRS were both inversely associated with MetS incidence, while neither GRS significantly predicted MI risk (Figure 2B). Sensitivity analyses, including cancer patients, showed consistent patterns (Figure S4A,B).

### 3.5. Gene–Diet Interactions and Genetic Susceptibility to WOE

WOE prevalence decreased with higher adherence to healthy dietary patterns across all GRS categories (Figure S4, Table 4). Significant GRS–diet interactions emerged for KBD (*p*-interaction = 0.042) and HEI (*p*-interaction = 0.031), with high dietary adherence reducing excess WOE risk by 16% and 30%, respectively, among high-GRS individuals (Figure S5A,B). Conversely, high coffee consumption amplified genetic risk in high-GRS individuals (*p*-interaction = 0.043; Figure S5C), while the GRS × flavonoid interaction was not significant (*p* = 0.14; Table 4). These findings suggest that overall diet quality, rather than specific anti-inflammatory nutrients, modifies genetic susceptibility to immune activation.

### 3.6. Immune Phenotype–Diet Interactions in Incident MetS

**Figure 3:**
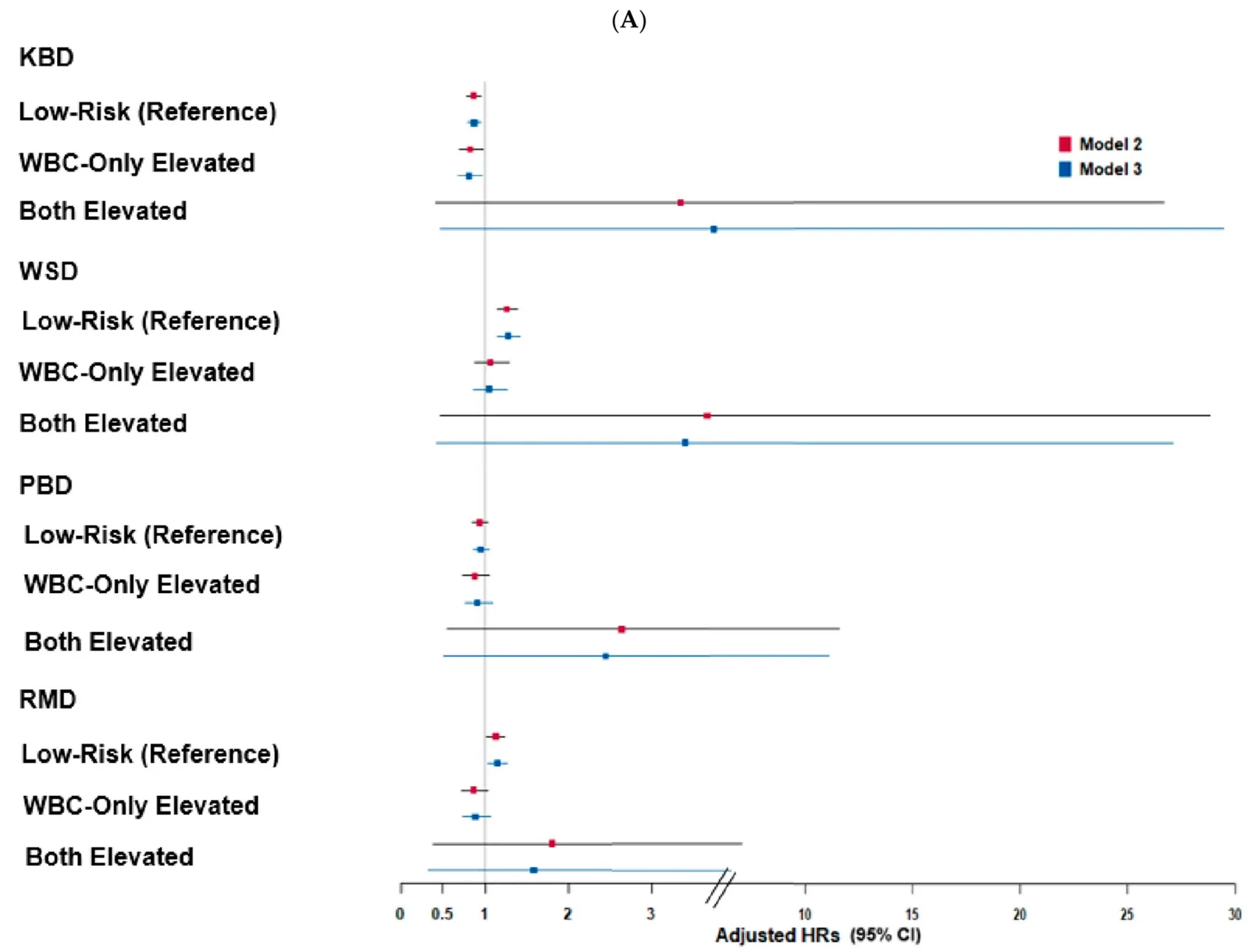

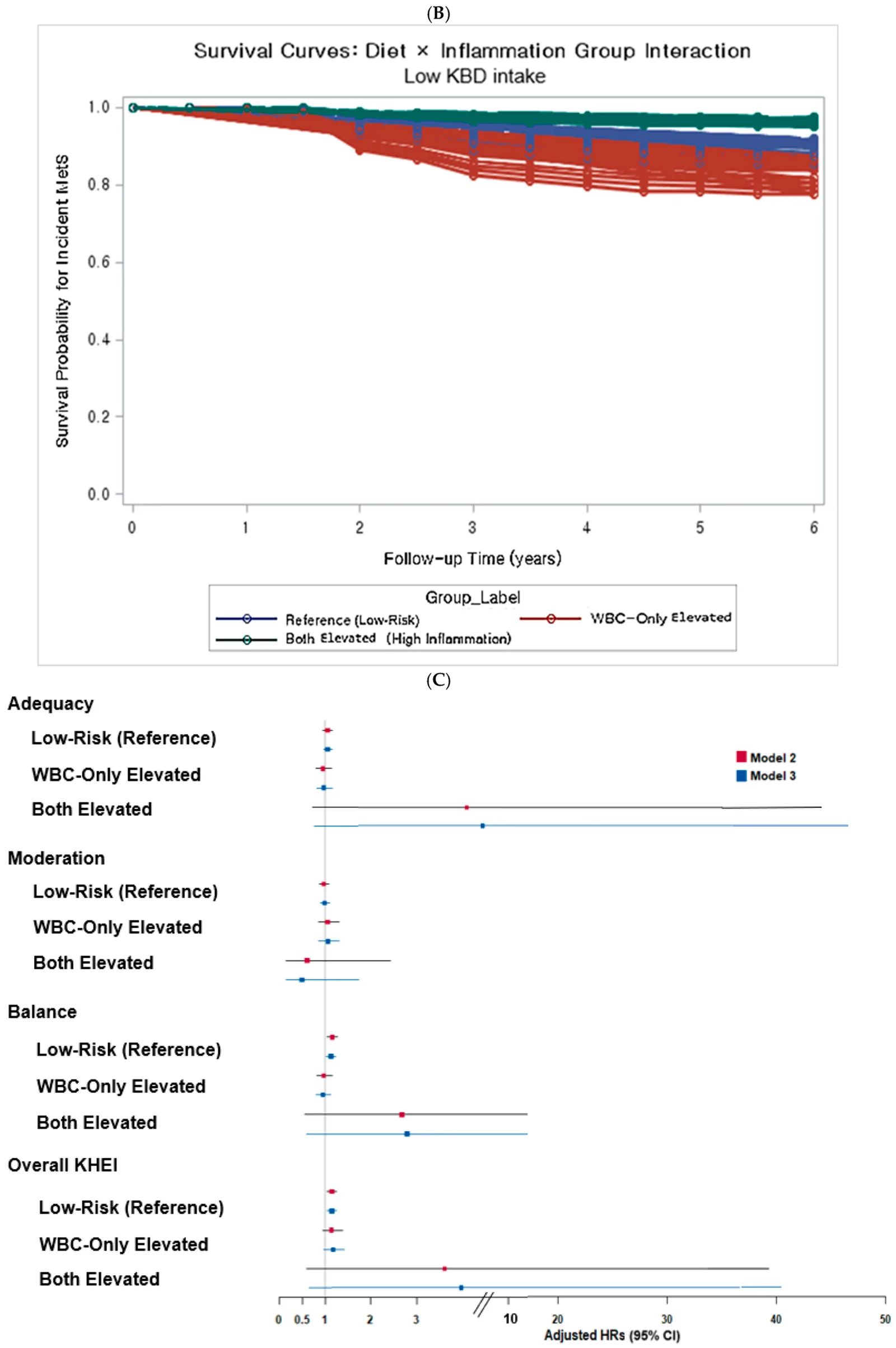
Associations between immune phenotypes and incident metabolic syndrome (MetS) according to dietary patterns and diet quality. (**A**) Adjusted hazard ratios (HRs) and 95% confidence intervals (CIs) for incident MetS by immune phenotype across four dietary patterns: Korean-style balanced diet (KBD), Western-style diet (WSD), egg-and-dairy-rich diet (EDR), and rice-main diet (RMD). (**B**) Kaplan–Meier survival curves for incident MetS stratified by immune phenotype within the highest diet quality stratum over a 5-year follow-up period. (**C**) Adjusted HRs and 95% CIs for incident MetS by immune phenotype across Healthy Eating Index (HEI) components (adequacy, moderation, balance, and overall HEI). WBC, white blood cell; CRP, C-reactive protein; WOE, WBC-Only Elevated; models adjusted for age, sex, residence area, follow-up period, education, income, BMI, energy intake, exercise, smoking, and alcohol consumption.

Dietary patterns modified associations between immune phenotypes and incident MetS (Figure 3A,B). WOE showed elevated MetS risk across all dietary patterns compared to Low Risk, with Both Elevated demonstrating the highest risk; HRs attenuated but remained elevated after full covariate adjustment (Figure 3A). Higher HEI and KBD scores were associated with lower MetS incidence within each immune phenotype group (Figure 3B), and Kaplan–Meier curves confirmed that cumulative MetS incidence was highest in Both Elevated and lowest in Low Risk, with WOE showing intermediate risk that became more pronounced among participants with lower dietary quality (Figure 3C). Sensitivity analyses yielded consistent results (Figure S6A–C).

## 4. Discussion

The present prospective study demonstrated that elevated WBC despite normal CRP independently predicted incident MetS and MI over a mean follow-up of five years in a large Korean cohort. Importantly, this immune profile, characterized by leukocyte-predominant activation without a concomitant hepatic acute-phase response, exhibited distinct genetic susceptibility, which was replicated in the UK Biobank, and healthy dietary patterns attenuated genetic predisposition to this phenotype. Together, these findings suggest that WBC provides clinically meaningful prognostic information beyond CRP alone, with implications for cardiometabolic risk stratification and dietary prevention.

The biological mechanism underlying WOE-associated cardiometabolic risk likely reflects a distinct pathway of innate immune activation characterized by sustained leukocyte mobilization without triggering the hepatic acute-phase response. Under normal inflammatory conditions, IL-6 stimulates hepatic CRP synthesis as part of the acute-phase response [35]. However, the WOE phenotype suggests persistent low-grade innate immune activation that drives leukocyte mobilization, particularly granulopoiesis and neutrophil mobilization, without reaching the threshold required for substantial CRP elevation [36]. This pattern of chronic subclinical leukocyte activation may promote endothelial dysfunction through direct leukocyte-endothelial interactions [37], contribute to insulin resistance via pro-inflammatory cytokine release from activated leukocytes [38], and accelerate adipose tissue inflammation and oxidative stress, collectively driving MetS and atherosclerotic progression [36]. Supporting this biological interpretation, γ-GTP concentrations were elevated in the WOE group without a corresponding elevation in aminotransferase levels, a pattern previously associated with subclinical hepatocellular metabolic stress and oxidative injury rather than overt hepatic inflammation [39]. These findings suggest that WBC captures a biologically distinct dimension of immune activation that complements rather than duplicates CRP.

The GWAS findings identified variants at immune-regulatory loci on 17q21.1 and 6p21.3, providing direct genetic evidence for the biological plausibility of this immune profile. At 17q21.1, CSF3 encodes granulocyte colony-stimulating factor, a key regulator of granulopoiesis and neutrophil mobilization [40,41,42,43], while Gasdermin A (GSDMA) mediates pyroptosis, a form of inflammatory cell death. These findings suggest that both leukocyte production and inflammatory cell turnover contribute to this phenotype [44]. At 6p21.3, HLA-C and HLA-DQA1 govern antigen presentation and T-cell activation [45], implicating chronic perturbation of adaptive immune recognition alongside innate immune dysregulation. The involvement of both innate (CSF3, GSDMA) and adaptive (HLA-C, HLA-DQA1) immune regulatory genes suggests that WOE reflects coordinated dysregulation across multiple immune pathways rather than a single inflammatory mechanism. The consistent replication of GRS associations in the UK Biobank supports the robustness and cross-ethnic generalizability of these genetic findings. Interestingly, high GRS was inversely associated with MetS incidence in both KoGES and UK Biobank cohorts, despite positive associations with WOE prevalence. This apparently paradoxical finding may reflect pleiotropic effects of the identified variants, particularly at 17q21.1 and 6p21.3, whereby genetic determinants of leukocyte mobilization simultaneously activate immune resolution pathways or enhance metabolic immune surveillance to attenuate downstream metabolic dysfunction [46]. Alternatively, this pattern may indicate that genetically elevated WBC represents a qualitatively different immune state from environmentally driven WBC elevation, with distinct downstream metabolic consequences. Further functional studies are needed to clarify these mechanisms.

Healthy dietary patterns, particularly KBD and higher HEI scores, attenuated genetic predisposition to WOE among individuals with high GRS. This finding is consistent with evidence that high-quality dietary patterns reduce oxidative stress and suppress NF-κB-mediated inflammatory signaling through fiber and micronutrient-rich food components [47]. Furthermore, plant-based and fermented foods characteristic of KBD modulate gut microbial composition, improving gut barrier integrity and reducing endotoxemia-driven systemic immune activation [48]. Importantly, protective effects were associated with overall dietary quality rather than individual nutrients, supporting the concept that synergistic dietary patterns exert greater biological effects than isolated dietary components [49]. This pattern is particularly relevant in the context of precision nutrition, where individual genetic profiles may determine differential responses to dietary interventions, suggesting that recommendations tailored to genetic susceptibility may be more effective than population-wide dietary guidelines for reducing cardiometabolic risk in individuals with subclinical leukocyte activation. In contrast, higher coffee consumption amplified genetic susceptibility to WOE, potentially reflecting inter-individual variation in caffeine and chlorogenic acid metabolism mediated by CYP1A2 polymorphisms [50,51]. However, because coffee intake was not consistently associated with MetS or MI risk, this finding requires confirmation in future studies. The absence of significant dietary pattern interactions for MI is consistent with prior evidence that leukocyte activation contributes more strongly to early metabolic dysfunction than to later cardiovascular events [52], reflecting different stages along the cardiometabolic disease continuum.

From a clinical perspective, individuals with normal CRP are often considered to have a relatively low inflammatory burden. However, 58.8% of this Korean cohort exhibited elevated WBC despite normal CRP and remained at significantly increased cardiometabolic risk; this subgroup would be systematically missed by CRP-only screening. This consideration is particularly relevant in East Asian populations, where baseline CRP concentrations are characteristically lower than in Western populations [53], further limiting CRP sensitivity. Because WBC is inexpensive, routinely available, and already included in standard complete blood count panels, joint assessment of WBC and CRP represents a practical and cost-neutral approach to improving early identification of high-risk individuals. Furthermore, the attenuation of genetic susceptibility by healthy dietary patterns, particularly KBD and high overall diet quality, suggests that dietary modification represents an accessible strategy for reducing cardiometabolic risk in genetically susceptible individuals with WOE, providing a rationale for precision nutrition approaches in this population.

This study extends previous evidence linking elevated WBC to cardiometabolic disease by demonstrating that prognostic significance persists even in the absence of CRP elevation. By integrating prospective epidemiological analyses with GWAS, cross-ethnic replication, and gene–diet interaction analyses, we provide evidence that this immune profile has a measurable genetic basis modifiable by dietary patterns. These findings highlight the potential value of combining routine inflammatory biomarkers with genetic and dietary information to improve cardiometabolic risk stratification. Future randomized controlled trials are needed to determine whether dietary interventions tailored to immune and genetic profiles can meaningfully reduce cardiometabolic risk in individuals with the WOE phenotype.

Several limitations warrant consideration. First, the observational design precludes causal inference; Mendelian randomization approaches using genetic variants as instrumental variables represent an important future direction for establishing causality. Second, WBC and CRP were measured only at baseline, precluding evaluation of temporal changes in immune status during follow-up; repeated measurements would provide greater insight into phenotype stability and disease progression. Third, residual confounding from unmeasured or imprecisely measured factors cannot be excluded despite adjustment for major covariates. Fourth, dietary intake assessment using SQFFQ and 24 h recall [27], although validated, is subject to reporting bias and measurement error. Fifth, the relatively small number of incident MI cases may have limited statistical power for modest associations. Finally, because the discovery cohort consisted primarily of East Asian participants, caution is warranted when generalizing genetic findings to other ethnic populations, although UK Biobank replication supports the robustness of identified loci across populations.

## 5. Conclusions

This study demonstrates that the WOE phenotype is a clinically meaningful predictor of incident MetS and MI, one that would be missed by CRP-based screening alone. The genetic variants identified through GWAS and replicated in the UK Biobank support a distinct biological basis for WOE susceptibility beyond that captured by conventional inflammatory markers. Importantly, the gene–diet interactions identified here indicate that dietary patterns can attenuate genetic susceptibility to WOE, suggesting that cardiometabolic risk in genetically susceptible individuals is not fixed but modifiable. Together, these findings support integrating WBC-based screening, genetic risk assessment, and dietary pattern information into precision prevention strategies, particularly in East Asian populations, where lower baseline CRP concentrations may cause individuals with elevated WBC to be misclassified as Low Risk.

## Figures and Tables

**Table 1:** Baseline characteristics of participants by immune phenotype at the first recruitment.

	Low Risk (N-WBC/N-CRP) (n = 21,828)	Acute Inflammation (N-WBC/H-CRP) (n = 154)	WBC-Only Elevated (H-WBC/N-CRP) (n = 32,187)	Both Elevated (H-WBC/H-CRP) (n = 575)
WBC (×10^9^/L)	4.87 ± 0.81 ^c^	4.88 ± 0.86 ^c^	7.42 ± 1.22 ^b^	8.36 ± 1.95 ^a^***
CRP (mg/dL)	0.09 ± 0.10 ^d^	2.08 ± 1.57 ^b^	0.11 ± 0.13 ^c^	2.22 ± 1.89 ^a^***
Age (years)	53.9 ± 7.91 ^c^	55.7 ± 8.18 ^b^	53.5 ± 8.16 ^c^	55.2 ± 8.04 ^a^**
Gender (men; N, %)	6328 (28.9)	49 (32.2)	11,940 (37.1)	285 (49.4) ***
BMI (kg/m^2^)	23.6 ± 2.82 ^b^	24.4 ± 2.91 ^a^	24.1 ± 2.86 ^a^	24.5 ± 3.31 ^a^**
Education (N, %) <high school>	4298 (19.7)	37 (24.3)	6631 (20.6)	135(23.5) *
High school	16,044 (73.5)	102 (66.4)	23,271 (72.3)	395 (68.6)
>high school	1480 (6.78)	14 (9.29)	2295 (7.13)	46 (7.96)
Metabolic syndrome (N, %)	2685 (12.3)	29 (19.0)	6695 (20.8)	131 (22.7) ***
Myocardial infarction (N, %)	587 (2.69)	5 (3.14)	1094 (3.4)	18 (3.15) ***
Cancer (N, %)	838 (3.84)	7 (4.40)	1053 (3.27)	36 (6.31) **
Waist circumferences (cm)	79.5 ± 8.54 ^c^	81.6 ± 8.45 ^ab^	81.6 ± 8.57 ^b^	84.2 ± 8.86 ^a^***
HDL (mg/dL)	55.0 ± 13.1 ^a^	52.7 ± 14.0 ^b^	52.7 ± 12.6 ^b^	49.7 ± 12.4 ^c^***
TG (mg/dL)	116 ± 75.6 ^b^	105 ± 68.1 ^c^	129 ± 85.4 ^a^	122 ± 79.4 ^a^***
LDL (mg/dL)	119 ± 31.8 ^b^	115 ± 30.8 ^ab^	117 ± 34.1 ^b^	121 ± 32.4 ^ab^**
Glucose (mg/dL)	93.4 ± 16.9	99.6 ± 27.3	95.4 ± 20.7	100 ± 27.0
SBP (mmHg)	122 ± 14.5 ^b^	122 ± 14.3 ^ab^	123 ± 15.1 ^a^	123 ± 14.8 ^a^***
DBP (mmHg)	75.0 ± 9.53 ^b^	75.7 ± 9.43 ^b^	76.6 ± 9.74 ^ab^	76.9 ± 9.63 ^a^***
γ-GTP (IU/L)	27.3 ± 32.4 ^c^	33.5 ± 32.7 ^b^	31.0 ± 32.2 ^b^	43.0 ± 38.6 ^a^***
AST (IU/dL)	23.0 ± 11.4 ^b^	24.4 ± 12.5 ^a^	24.1 ± 13.5 ^a^	24.6 ± 13.5 ^a^***
ALT (IU/dL)	20.8 ± 15.8 ^c^	22.0 ± 14.0 ^a^	23.4 ± 18.2 ^a^	25.1 ± 21.7 ^b^**
Homocysteine (μM)	8.53 ± 3.33 ^c^	8.58 ± 2.64 ^c^	9.36 ± 3.55 ^b^	9.94 ± 3.70 ^a^***

Means ± standard deviation or numbers (percentages) are presented. Immune phenotypes were categorized into four groups based on white blood cell (WBC) count (cutoff: 6.2 × 10^9^/L) and C-reactive protein (CRP) level (cutoff: 1 mg/dL). Abbreviations: N-, normal; H-, high; WBC, white blood cells; CRP, C-reactive protein; MI, myocardial infarction; CVD, cardiovascular disease; BMI, body-mass index; HDL, high-density lipoprotein; LDL, low-density lipoprotein; TG, triglyceride; SBP, systolic blood pressure; DBP, diastolic blood pressure; γ-GTP, Gamma-glutamyl transferase; AST, aspartate aminotransferase; ALT, alanine aminotransferase. * *p* < 0.05, ** *p* < 0.01, and *** *p* < 0.001 indicate significant differences among immune phenotypes. Different superscript letters (a, b, c, d) denote significant pairwise differences based on Tukey’s test (*p* < 0.05).

**Table 2:** Nutrient intake, Healthy Eating Index (HEI), and dietary patterns in the first recruitment.

	Low Risk (N-WBC/N-CRP) (n = 21,828)	Acute Inflammation (N-WBC/H-CRP) (n = 154)	WBC-Only Elevated (H-WBC/N-CRP) (n = 32,187)	Both Elevated (H-WBC/H-CRP) (n = 575)
Energy (EER %)	99.0 ± 30.5 ^a^	95.0 ± 33.5 ^ab^	99.1 ± 33.2 ^a^	95.4 ± 32.4 ^b^***
CHO (En%)	72.0 ± 6.83 ^a^	71.7 ± 7.67 ^ab^	71.8 ± 6.98 ^b^	72.0 ± 7.03 ^ab^**
Protein (g/kg body weight)	0.46 ± 0.29	0.43 ± 0.32	0.46 ± 0.31	0.43 ± 0.28
Fat (En%)	13.7 ± 5.29	13.8 ± 5.83	13.8 ± 5.39	13.6 ± 5.49
Dietary inflammation index	−17.8 ± 15.2 ^ab^	−16.9 ± 11.9 ^a^	−18.4 ± 16.8 ^b^	−17.6 ± 15.9 ^ab^**
Flavonoids (mg/d)	43.4 ± 33.1 ^a^	43.4 ± 33.6 ^ab^	45.1 ± 38.8 ^b^	42.0 ± 42.5 ^ab^***
V-C (mg/d)	106 ± 65.0 ^ab^	101 ± 63.4 ^b^	110 ± 73.5 ^a^	103 ± 68.6 ^b^***
V-E (mg/d)	8.03 ± 4.20 ^b^	7.71 ± 4.31 ^b^	8.28 ± 4.71 ^a^	8.00 ± 4.60 ^ab^***
V-D (μg/d)	8.62 ± 5.14 ^b^	8.32 ± 6.36 ^ab^	8.95 ± 5.78 ^a^	8.69 ± 5.92 ^ab^***
Fiber (g/d)	14.3 ± 8.59 ^b^	13.7 ± 6.54 ^ab^	15.6 ± 10.1 ^a^	15.1 ± 9.57 ^ab^***
Calcium (mg/d)	477 ± 331 ^b^	459 ± 300 ^ab^	487 ± 337 ^a^	469 ± 344 ^ab^**
Na (g/day)	2.39 ± 13.0 ^b^	2.42 ± 11.6 ^ab^	2.53 ± 15.0 ^a^	2.47 ± 13.9 ^ab^***
Coffee (g/day)	3.57 ± 3.00	3.54 ± 3.17	3.51 ± 3.06	3.51 ± 3.09
Glycemic index	48.3 ± 9.58 ^b^	49.2 ± 10.3 ^ab^	49.4 ± 10.0 ^a^	50.0 ± 10.5 ^a^***
KBD (N, %)	7028 (32.2)	46 (30.1)	10621(33.0)	173 (30.0)
WSD (N, %)	6723 (30.8)	50 (32.2)	12199 (37.9)	221 (38.5) ***
PBD (N, %)	7264 (33.3)	47 (30.7)	9386(29.2)	163 (28.4) ***
RMD (N, %)	7138 (32.7)	50 (32.4)	9965 (31.0)	214 (37.2) ***
Adequacy scores in HEI	35.4 ± 8.38 ^a^	35.0 ± 8.48 ^ab^	35.0 ± 8.69 ^b^	34.0 ± 8.99 ^c^***
Moderation scores in HEI	43.1 ± 8.35 ^a^	43.1 ± 8.44 ^ab^	42.4 ± 8.80 ^b^	42.6 ± 8.78 ^ab^***
Balance scores in HEI	13.3 ± 6.78	12.9 ± 7.02	13.4 ± 6.94	13.1 ± 7.43
Overall HEI scores	91.9 ± 11.1 ^a^	90.9 ± 11.7 ^ab^	90.8 ± 11.3 ^b^	89.7 ± 12.2 ^c^***
Alcohol intake (g/day)	13.1 ± 4.24 ^b^	11.1 ± 2.62 ^b^	14.6 ± 2.98 ^b^	18.1 ± 3.09 ^a^***
Physical activity (min/day)	18.3 ± 10.5 ^a^	17.7 ± 14.7 ^ab^	15.5 ± 9.36 ^b^	16.0 ± 9.44 ^b^***
Non-Smoker (N, %)	16,954 (77.7)	117 (76.0)	22,759 (70.7)	367 (63.8) ***
Former smoker	3388 (15.5)	29 (18.6)	4992 (15.5)	111 (19.4)
Smoker	1369 (6.3)	8 (4.9)	4145 (12.9)	93 (16.2)

Means ± standard deviation or numbers (percentages) are presented. Immune phenotypes were categorized into four groups based on white blood cell (WBC) count (cutoff: 6.2 × 10^9^/L) and C-reactive protein (CRP) level (cutoff: 1 mg/dL). Abbreviations: N-, normal; H-, high; EER%, percentage of estimated energy requirement (EER); En%, percentage of daily energy intake; CHO, carbohydrate; KBD, Korean-style balanced diet; WSD, Western-style diet; PBD, plant-based diet; RMD, rice-main diet; HEI, Healthy Eating Index. ** *p* < 0.01 and *** *p* < 0.001 indicate significant differences among immune phenotypes. Different superscript letters (a, b, c) denote significant pairwise differences according to Tukey’s test (*p* < 0.05).

**Table 3:** Characteristics of genetic variants associated with the WBC-Only Elevated group based on the Low-Risk group.

			KOGES										UK BIOBANK				
CHR	SNP	BP	Risk	A1	A2	OR	SD	*p*	Gene Names	Location	MAF	HWE	A1	REF	OR	SD	*p*
2	rs1260326	27730940	C	C	T	1.12	4.08	1.19 × 10^−9^	*GCKR*	Missense	0.4498	0.0497	T	T	0.956	3.62	7.13 × 10^−17^
2	rs76260229	136511617	G	T	G	1.190	5.15	1.73 × 10^−13^	*UBXN4*	3′-UTR	0.1794	0.7366	T	G	0.831	16.97	8.13 × 10^−4^
2	rs3754689	136590746	C	T	C	1.112	4.08	3.12 × 10^−8^	*LCT*	Missense	0.3573	0.6339	T	C	1.024	5.23	2.12 × 10^−8^
2	rs309133	136751842	A	C	A	1.124	4.08	9.63 × 10^−10^	*DARS1*	3′-UTR	0.1832	0.7616	C	A	1.001	5.3	8.94 × 10^−3^
6	rs887464	31145920	T	T	C	1.112	4.08	1.35 × 10^−8^	*POU5F1*	Intron	0.4288	0.781	T	C	0.907	3.56	1.05 × 10^−75^
6	rs1050428	31239543	T	T	C	1.183	5.58	2.05 × 10^−10^	*HLA-C*	Missense	0.1059	0.8445	T	C	1.165	5.1	1.31 × 10^−88^
6	rs10093	32609173	C	C	G	1.113	3.86	5.77 × 10^−9^	*HLA-DQA1*	Missense	0.476	0.7658	G	C	0.893	3.89	1.75 × 10^−84^
7	rs41348	28716848	A	G	A	1.110	4.08	3.17 × 10^−8^	*CREB5*	Intron	0.3806	0.7593	G	A	0.942	3.62	6.58 × 10^−28^
7	rs445	92408370	T	T	C	1.176	4.25	2.78 × 10^−16^	*CDK6*	Intron	0.3273	0.8882	T	C	0.851	5.84	2.22 × 10^−76^
17	rs56030650	38131187	C	A	C	1.177	3.95	9.62 × 10^−19^	*GSDMA*	Missense	0.4995	0.2832	A	C	0.858	3.56	7.35 × 10^−184^
17	rs7021	38153875	T	A	T	1.190	3.95	3.67 × 10^−21^	*PSMD3*	3′-UTR	0.4668	0.1765	A	T	0.844	3.62	1.56 × 10^−213^
17	rs25645	38173143	A	A	G	1.198	4.05	1.06 × 10^−21^	*CSF3*	3′-UTR	0.4121	0.6517	A	G	0.890	3.62	7.50 × 10^−102^
17	rs2302777	38179492	G	G	A	1.201	4.05	3.39 × 10^−22^	*MED24*	3′-UTR	0.4131	0.9322	G	A	0.890	3.69	3.01 × 10^−100^

Genome-wide significant variants are shown at *p* < 5 × 10^−8^. Models were adjusted for age, residential region, education, income, energy intake, drinking status, smoking status, and physical activity. The table includes chromosome (CHR), single nucleotide polymorphism (SNP), base-pair position (BP), minor allele (A1), major allele (A2), risk allele (Risk), odds ratio (OR), standard deviation (SD), *p*-value, nearest gene (gene names), genomic location (location), minor allele frequency (MAF), and Hardy–Weinberg equilibrium (HWE).

**Table 4:** Interaction between genetic risk score (GRS) and lifestyle factors for the WBC-Only Elevated group at the first recruitment.

	Low-GRS (n = 21,469)	Medium-GRS (n = 19,897)	High-GRS (n = 11,030)	*p*-Value for the Interaction of GRS and Lifestyles
KBD				0.0423
Low intake	1	1.171 (1.092–1.255)	1.477 (1.355–1.610)	
High intake	1	1.291 (1.163–1.433)	1.402 (1.251–1.612)	
Overall HEI				0.0307
Low score	1	1.257 (1.139–1.388)	1.512(1.384–1.652)	
High score	1	1.181 (1.099–1.268)	1.360 (1.211–1.546)	
Coffee				0.0431
Low intake	1	1.123 (1.016–1.240	1.361 (1.202–1.541)	
High intake	1	1.259 (1.170–1.349)	1.515 (1.389–1.652)	
Flavonoids				0.1435
Low intake	1	1.17 (1.091–1.254)	1.483 (1.361–1.617)	
High intake	1	1.294 (1.166–1.436)	1.403 (1.237–1.591)	

Adjusted odds ratios (ORs) and 95% confidence intervals (CIs) are shown for the association between lifestyle factors and the high WBC plus normal CRP phenotype across GRS tertiles (low, medium, high). The cutoffs for flavonoid intake, the Korean-style balanced diet (KBD), overall Healthy Eating Index (HEI) scores, and coffee intake were the 66th percentiles. Models were adjusted for age, residential region, education, income, energy intake, drinking status, smoking status, and physical activity.

## Data Availability

The data were included in the manuscript and Supplementary Information, but the analyzed data not included are available from the corresponding author on reasonable request.

## Supplementary Materials

The following supporting information can be downloaded at https://www.mdpi.com/article/10.3390/nu18183040/s1: Figure S1: Flowchart of participant selection from the 1st and 2nd recruitments in the Health Examinee Study (HEXA). Figure S2: Associations of WBC-Only Elevated (WOE) by white blood cells (WBC) and serum C-reactive protein (CRP) levels with metabolic syndrome (MetS) and myocardial infarction (MI) without participants having a cancer history. Figure S3: Characteristics of genetic variants of the WBC-Only Elevated (WOE; immune activation). Figure S4: Association of weighted genetic risk scores (GRS) with WBC-Only Elevated (WOE), metabolic syndrome (MetS), and myocardial infarction (MI) without participants having a cancer history. Figure S5: Prevalence of WBC-Only Elevated (WOE) stratified by weighted genetic risk scores (GRS) and dietary factors. Figure S6: Associations of WBC-Only Elevated (WOE) with metabolic syndrome (MetS) according to dietary patterns and diet quality without participants with a cancer history. Table S1: Adjusted means of nutrient intake, Healthy Eating Index (HEI), and dietary patterns in the 1st recruitment. Table S2: Genome-wide significant variants associated with high WBC and normal CRP compared with normal WBC and normal CRP in KoGES. Table S3: Linear associations of genome-wide significant variants identified by logistic GWAS with the high WBC and normal CRP phenotype in KoGES and UK Biobank.

## Abbreviations

WBC	White Blood Cell
CRP	C-Reactive Protein
MetS	Metabolic Syndrome
MI	Myocardial Infarction
CVD	Cardiovascular Disease
GWAS	Genome-Wide Association Study
PRS	Polygenic Risk Score
GRS	Genetic Risk Score
SNP	Single Nucleotide Polymorphism
UTR	Untranslated Region
HR	Hazard Ratio
OR	Odds Ratio
CI	Confidence Interval
KoGES	Korean Genome and Epidemiology Study
UKB	UK Biobank
KHEI	Korean Healthy Eating Index
EER	Estimated Energy Requirement
AST	Aspartate Aminotransferase
ALT	Alanine Aminotransferase
γ-GTP	Gamma-Glutamyl Transpeptidase
WOE	WBC-Only Elevation
KBD	Korean Balanced Diet
WSD	Western-Style Diet
PBD	Plant-Based Diet
RMD	Rice-Main Diet
