# Supplementary material for: Elevated White Blood Cell Counts Despite Normal C-Reactive Protein Independently Predict Metabolic Syndrome and Myocardial Infarction: Evidence for Genetic Susceptibility and Dietary Modification in a Prospective Cohort
Source: Nutrients. 2026 Sep 17;18(18):3040. doi: 10.3390/nu18183040 (PMC13610106; doi:10.3390/nu18183040)
Supplement: Supplementary file 1 [file nutrients-18-03040-s001.zip › nutrients-4507916-supplementary.pdf]

Supplementary Figures

Figure S1. Flowchart of participant selection from 1st and 2nd recruitments in Health Examinee Study (HEXA).

Initially, 173,195 participants (aged 40–75 years) were recruited in the 1st phase (2004–2013). After an over 5-year gap, 107,584 participants were excluded for not participating in the 2nd recruitment, and 10,660 were excluded due to incomplete data. Without missing serum CRP levels in the 1st recruitment, 54,941 were in the HEXA participants in the 1st recruitment. A 2nd recruitment (2012–2016) added 65,611 participants. Participants from both recruitments who had both CRP and WBC measurements totaled 54,856. After excluding 85 participants with no WBC measurement in the 2nd recruitment, this group was merged with 58,701 individuals from KoGES (Korean Genome and Epidemiology Study) for genetic variant measurement. Exclusion of 3,845 non-matching and 112 with incomplete genetic variants led to the final analysis of 54,744 participants. A sensitive analysis was also conducted on 52,814 participants by excluding the 1,934 individuals who had a cancer diagnosis.

Figure S2. Associations of WBC-Only Elevated (WOE) by white blood cells (WBC) and serum C-reactive protein (CRP) levels with metabolic syndrome (MetS) and myocardial infarction (MI) without the participants having a cancer history.

(A) Forest plot of adjusted hazard ratios (HRs) and 95% Confidence Intervals (CIs) for Inflammation Phenotypes without the participants having a cancer history.

(B) Kaplan-Meier survival curves for incident MetS. This plot illustrates the adjusted survival probability for developing MetS over a 6-year follow-up period across different inflammation risk groups without the participants having a cancer history.

(C) Kaplan-Meier survival curves for incident MI. This plot illustrates the adjusted survival probability for developing MI over a 5-year follow-up period across different

inflammation risk groups without the participants having a cancer history.

Low-Risk (normal WBC/normal CRP) group as the reference, WBC-Only Elevated (WOE; high WBC/normal CRP), and Both Elevated (High Inflammation; High WBC/High CRP). Model 1 adjusts for demographics, Model 2 for covariates in Model 1 plus metabolic factor, and Model 3 for covariates in Model 2 plus lifestyles.

Figure S3. Characteristics of genetic variants of the WBC-Only Elevated (WOE; immune activation)

(A) Manhattan plot of genetic variants by GWAS between the WOE (high WBC/normal CRP) and Low risk (normal WBC/normal CRP) groups.

(B) Q-Q plot of the genetic variants

(C) Connection pathway between the genes selected from the GWAS at $P< 5X10^{-8}$

GWAS was adjusted with Model 3 (age, gender, education, income, body mass index, energy and alcohol intake, smoking, and exercise). GWAS, genome-wide association study; WBC, white blood cells; CRP, C-reactive protein.

Figure S4. Association of weighted genetic risk scores (GRS) with WBC-Only Elevated (WOE), metabolic syndrome (MetS), and myocardial infarction (MI) without participants with a cancer history.

(A) Adjusted odds ratio (OR) and 95% confidence intervals (CI) of GRS with missense variants and 3'-UTR variants for WBC-Only Elevated (WOE; high-WBC/normal-CRP)

(B) Adjusted ORs and 95% CI of the GRS with Missense and 3'-UTR variants for MetS and MI

It examines the relationship between the GRS derived from the identified loci and the WOE (immune activation) phenotype, as well as the cardiometabolic outcomes. All associations are

adjusted using Model 3 (the fully adjusted model, accounting for demographic, metabolic, and lifestyle factors) to assess the independent effect of genetic risk on the cardiometabolic outcomes.

Figure S5. Prevalence of WBC-Only Elevated (WOE) stratified by weighted genetic risk scores (GRS) and dietary factors.

(A) Low and high scores of the overall healthy eating index (HEI) scores

(B) Low and high intake of the Korean-style balanced diet (KBD)

(C) Low and high intake of flavonoids intake

(D) Low and high intake of coffee intake

They present the prevalence of the WOE (high-WBC/normal-CRP) according to strata of the GRS with missense variants, further stratified by adherence to various dietary components (cutoffs at the 66th percentile).

Figure S6. Associations of WBC-Only Elevated (WOE) with metabolic syndrome (MetS) according to dietary patterns and diet quality without the participants with a cancer history.

(A) Forest plot of adjusted hazard ratios (HRs) and 95% Confidence Intervals (CIs) for WOE according to four dietary patterns in MetS incidence.

Adjusted HRs for MetS incidence stratified by four distinct composite dietary patterns, such as the Korean-style balanced diet (KBD), Western-style diet (WSD), plant-based diet (PBD), and Rice-main diet (RMD)

(B) Kaplan-Meier survival curves for incident MetS stratified by inflammation phenotype within the highest diet quality stratum.

This panel displays the adjusted survival probability for developing incident MetS over a 6-year follow-up period. The curves are specifically plotted for the highest diet quality stratum,

illustrating how the three inflammation groups—Low-Risk (normal WBC/normal CRP) group as the reference, WOE (high WBC/normal CRP), and Both Elevated (High Inflammation; High WBC/High CRP)—differ in their risk for MetS when adherence to the best diet is maximized.

(C) Forest plot of HRs and 95% CIs for WOE according to dietary quality by healthy eating index (HEI) in MetS incidence.

Adjusted HRs for MetS incidence stratified by components of diet quality, specifically the HEI, including adequacy, moderation, balance, and the overall HEI score.

Supplementary Tables

Table S1. Adjusted means of nutrient intake, healthy eating index (HEI), and dietary patterns in the 1st recruitment

Table S2. Genome-wide significant variants associated with high WBC and normal CRP compared with normal WBC and normal CRP in KoGES (logistic GWAS; $P < 5 \times 10^{-8}$; LD $r^2 < 0.001$)

Table S3. Linear associations of genome-wide significant variants identified by logistic GWAS with the high WBC and normal CRP phenotype in KoGES and UK Biobank

Figure S1

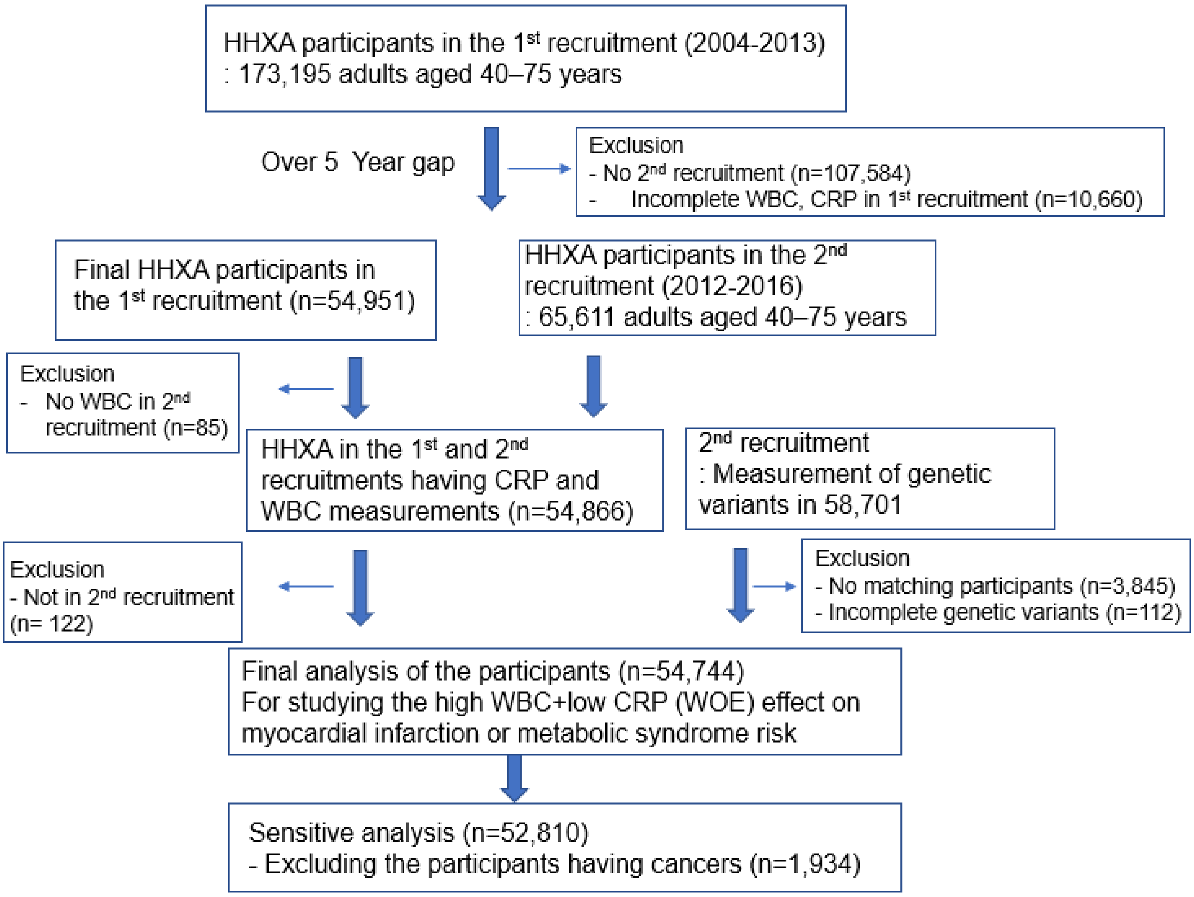

HHXA participants in the 1st recruitment (2004-2013)
: 173,195 adults aged 40–75 years
Over 5 Year gap
Exclusion
- No 2nd recruitment (n=107,584)
- Incomplete WBC, CRP in 1st recruitment (n=10,660)
Final HHXA participants in the 1st recruitment (n=54,951)
HHXA participants in the 2nd recruitment (2012-2016)
: 65,611 adults aged 40–75 years
Exclusion
- No WBC in 2nd recruitment (n=85)
HHXA in the 1st and 2nd recruitments having CRP and WBC measurements (n=54,866)
2nd recruitment
: Measurement of genetic variants in 58,701
Exclusion
- Not in 2nd recruitment (n= 122)
Exclusion
- No matching participants (n=3,845)
- Incomplete genetic variants (n=112)
Final analysis of the participants (n=54,744)
For studying the high WBC+low CRP (WOE) effect on myocardial infarction or metabolic syndrome risk
Sensitive analysis (n=52,810)
- Excluding the participants having cancers (n=1,934)

Figure S2A

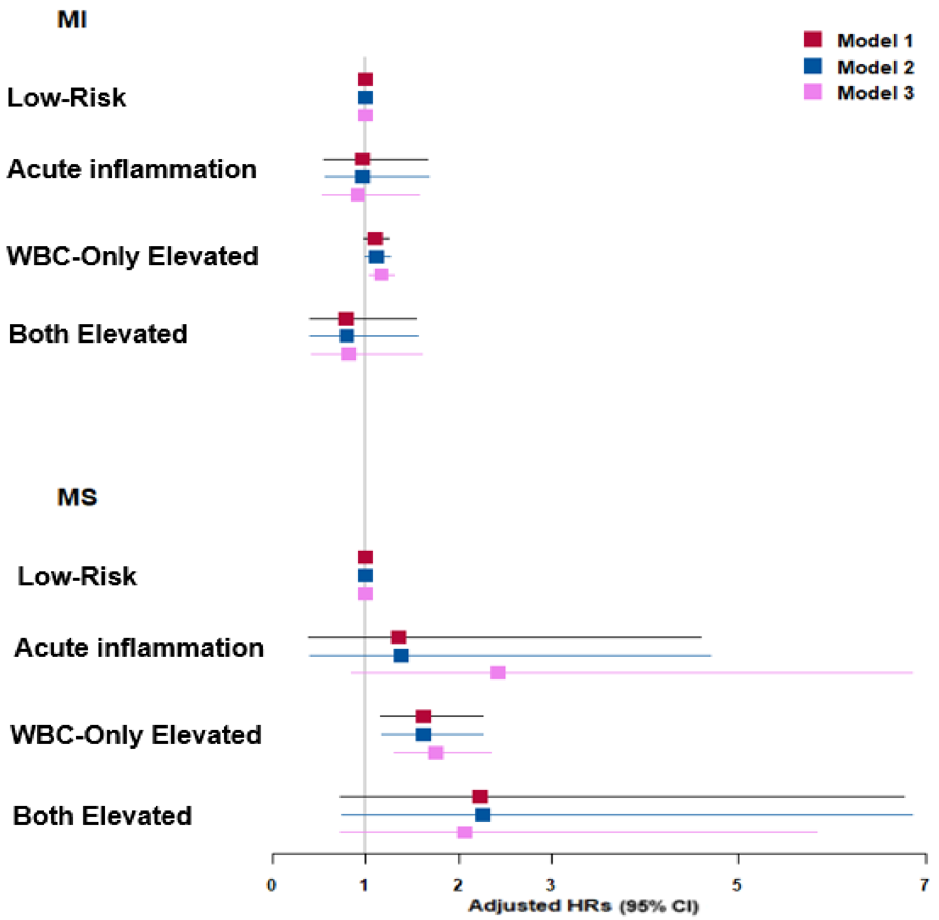
MI
Low-Risk
Acute inflammation
WBC-Only Elevated
Both Elevated
MS
Low-Risk
Acute inflammation
WBC-Only Elevated
Both Elevated
Model 1
Model 2
Model 3
0
1
2
3
5
7
Adjusted HRs (95% CI)

Figure S2B

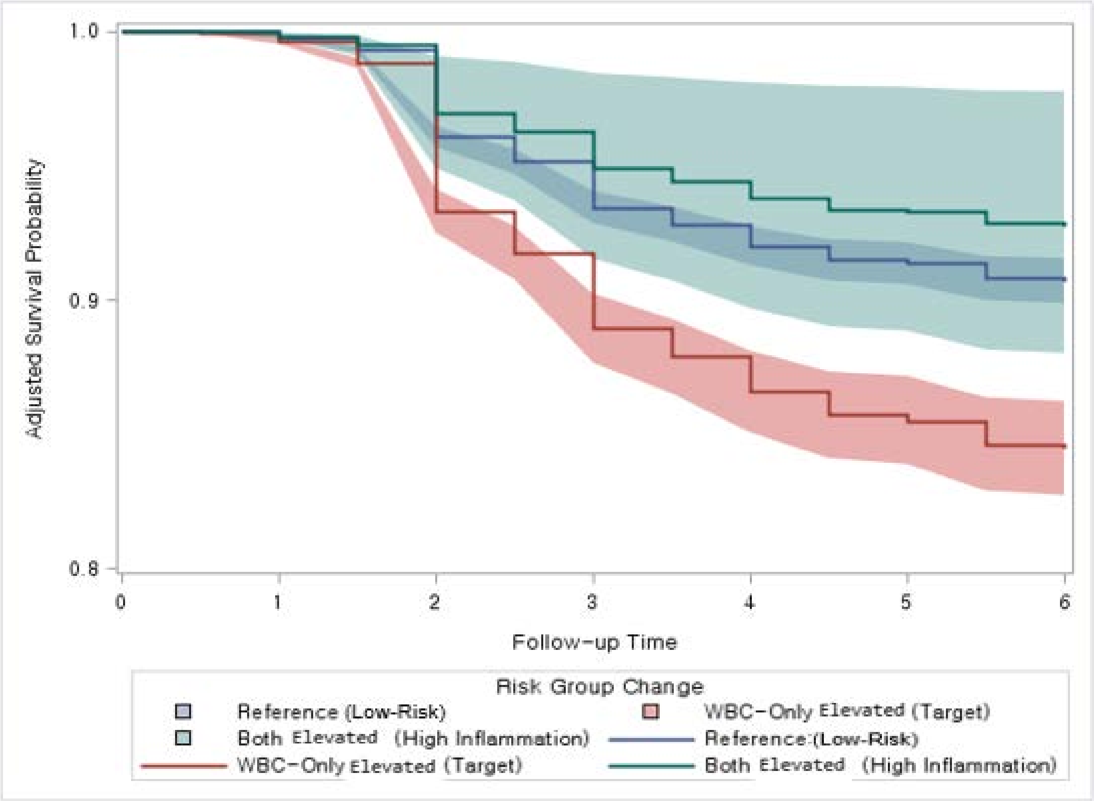

Figure S2C

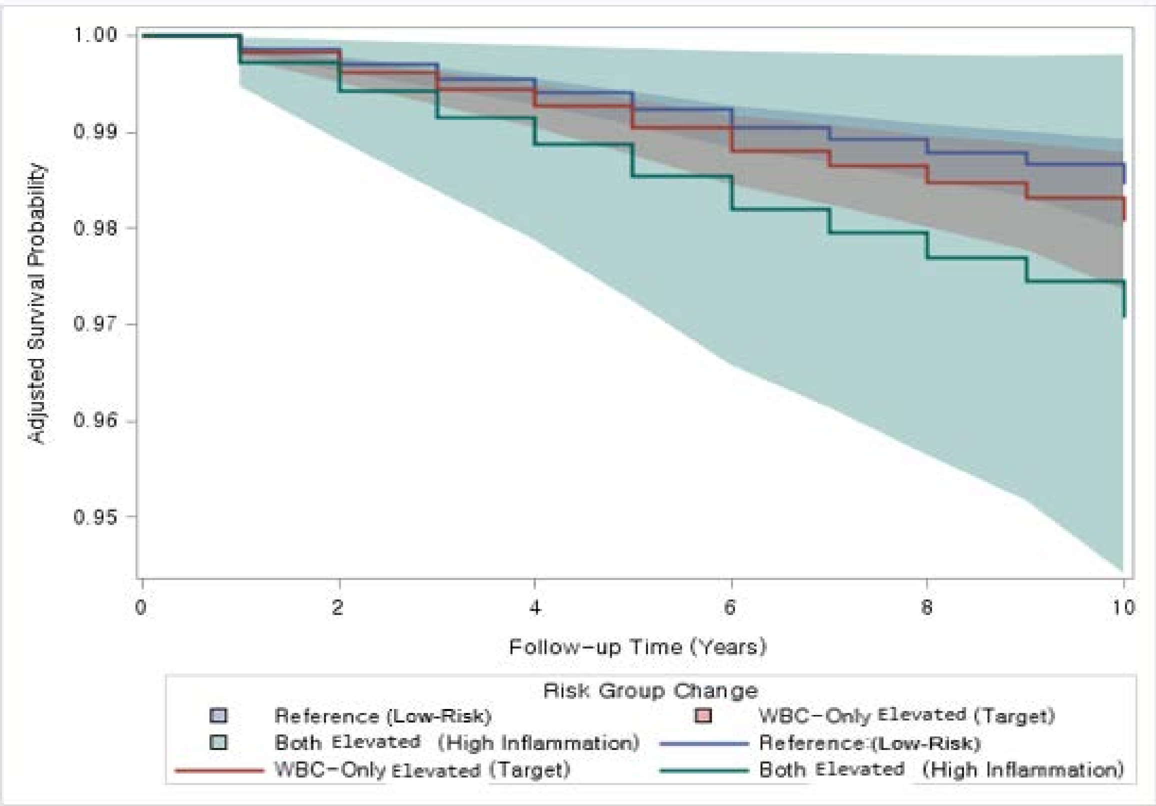

Figure S3A

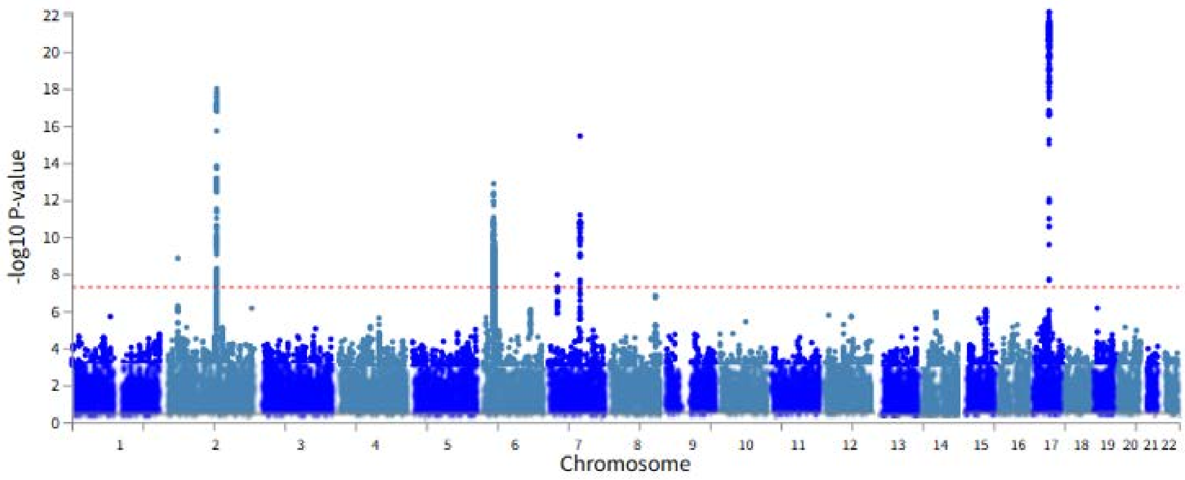

Figure S3B

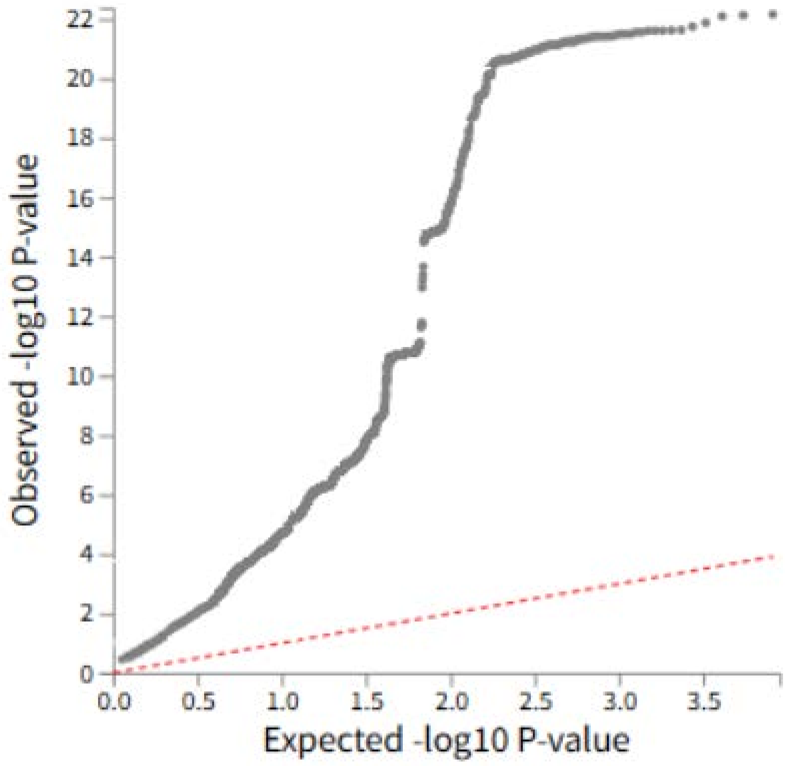

Figure S3C

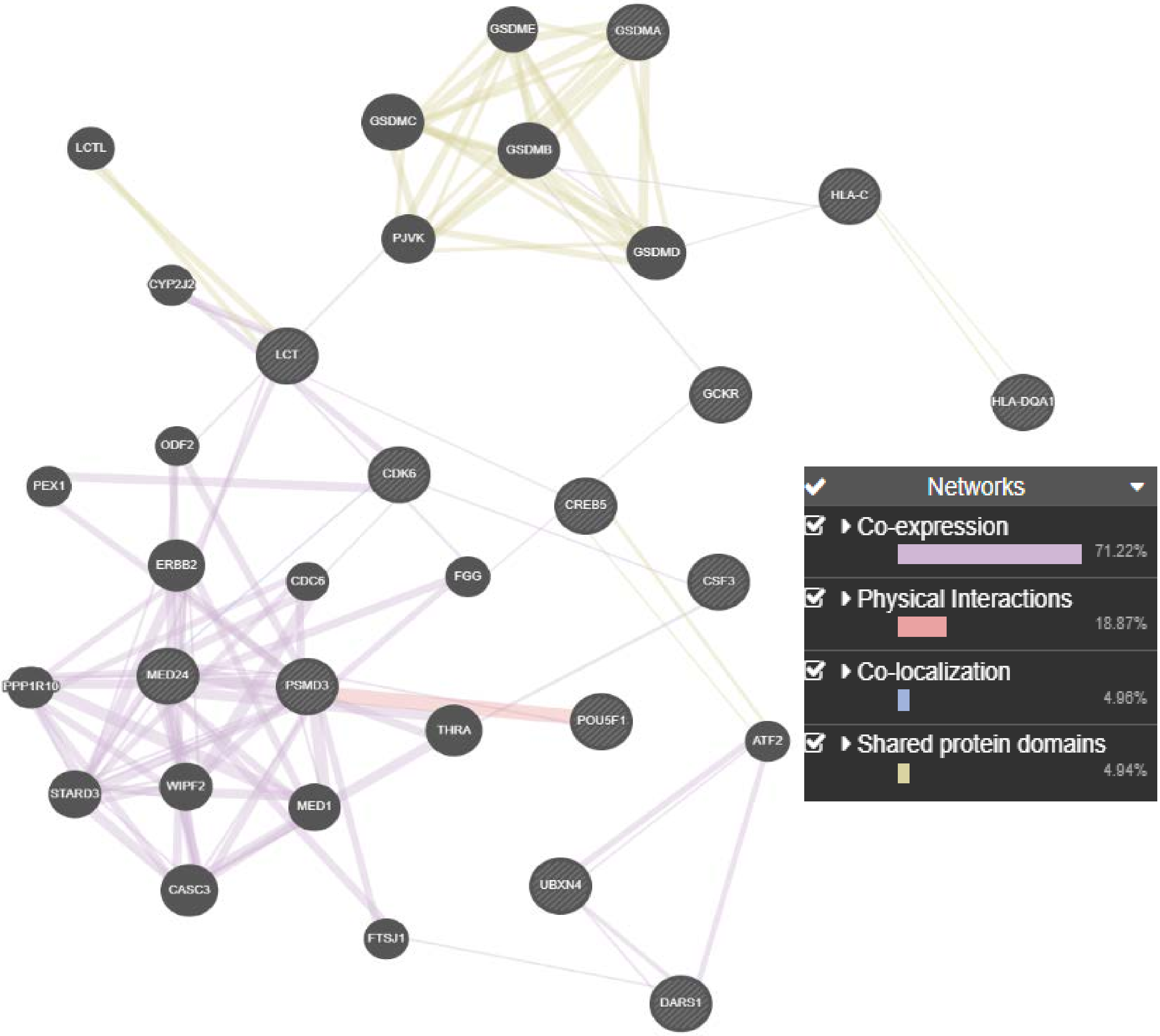
GSDME
GSDMA
GSDMC
GSDMB
LCTL
HLA-C
PJVK
GSDMD
CYP2J2
LCT
GCKR
HLA-DQA1
ODF2
PEX1
CDK6
CREB5
ERBB2
CDC6
FGG
CSF3
PPP1R10
MED24
PSMD3
THRA
POU5F1
ATF2
STARD3
WIPF2
MED1
CASC3
UBXN4
FTSJ1
DARS1
Networks
Co-expression
71.22%
Physical Interactions
18.87%
Co-localization
4.96%
Shared protein domains
4.94%

Figure S4A

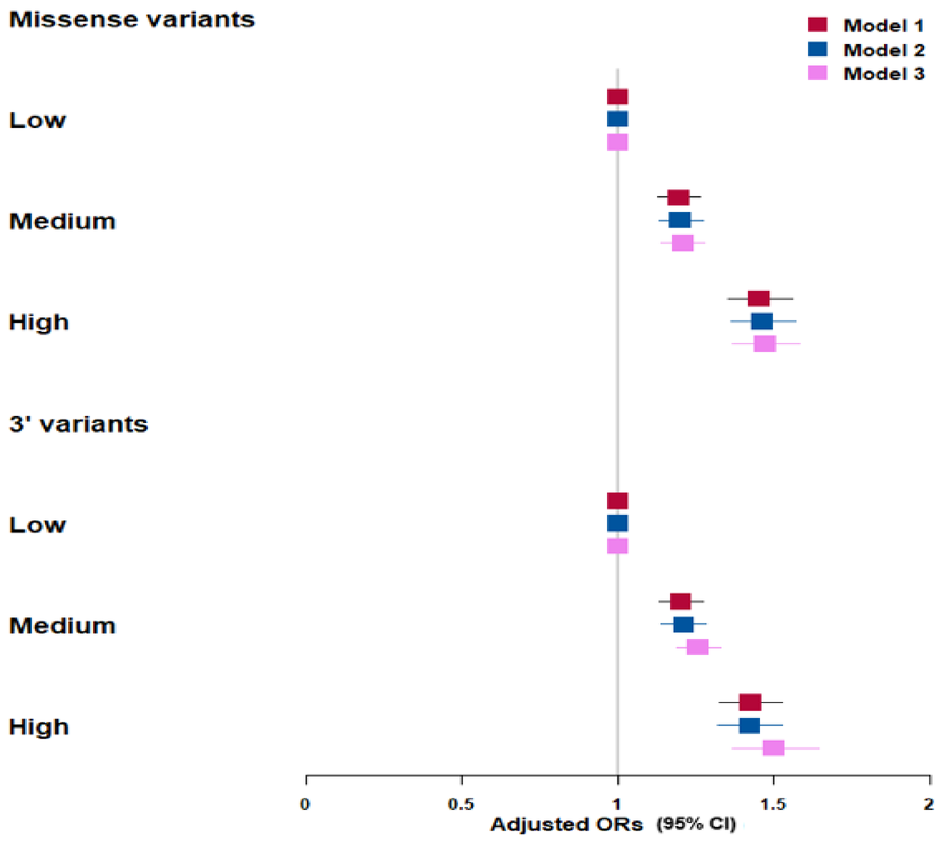

Figure S4B

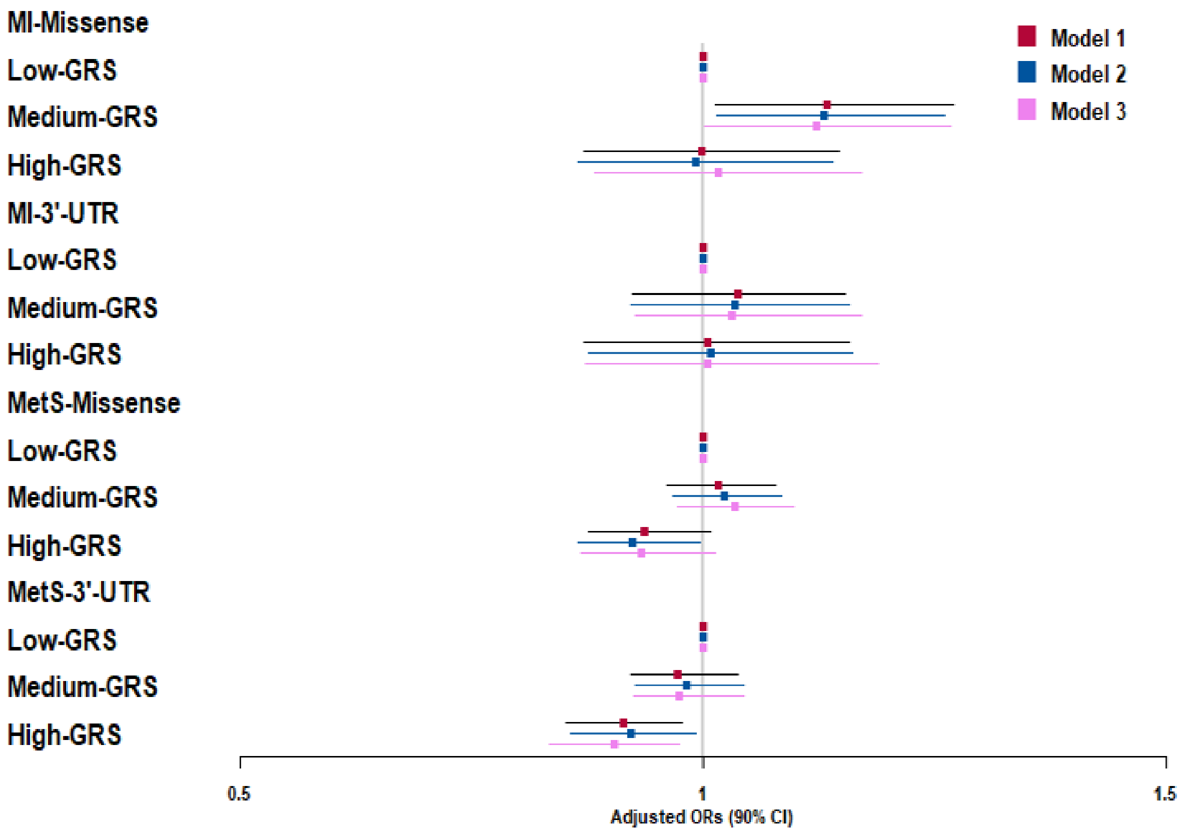

Figure S5

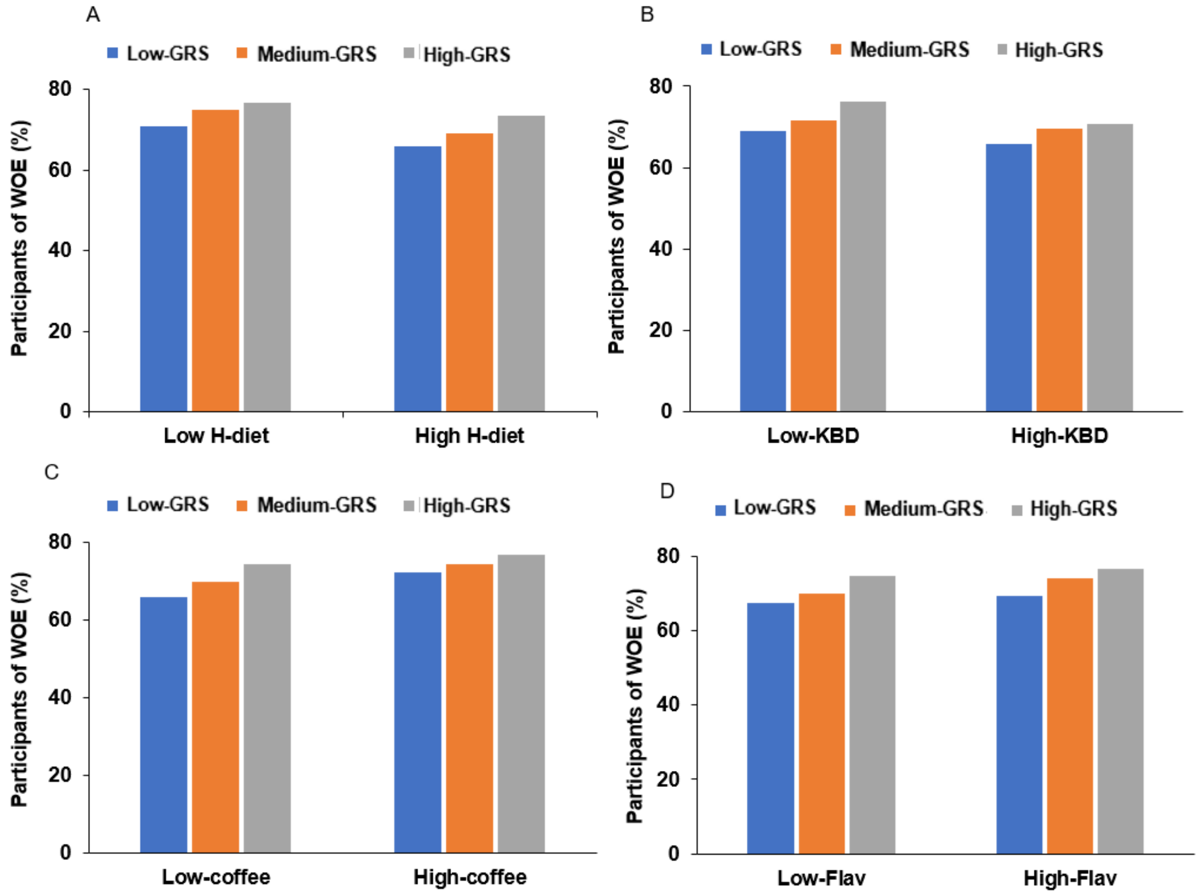
A
Low-GRS
Medium-GRS
High-GRS
Participants of WOE (%)
80
60
40
20
0
Low H-diet
High H-diet
B
Low-GRS
Medium-GRS
High-GRS
Participants of WOE (%)
80
60
40
20
0
Low-KBD
High-KBD
C
Low-GRS
Medium-GRS
High-GRS
Participants of WOE (%)
80
60
40
20
0
Low-coffee
High-coffee
D
Low-GRS
Medium-GRS
High-GRS
Participants of WOE (%)
80
60
40
20
0
Low-Flav
High-Flav

Figure S6A

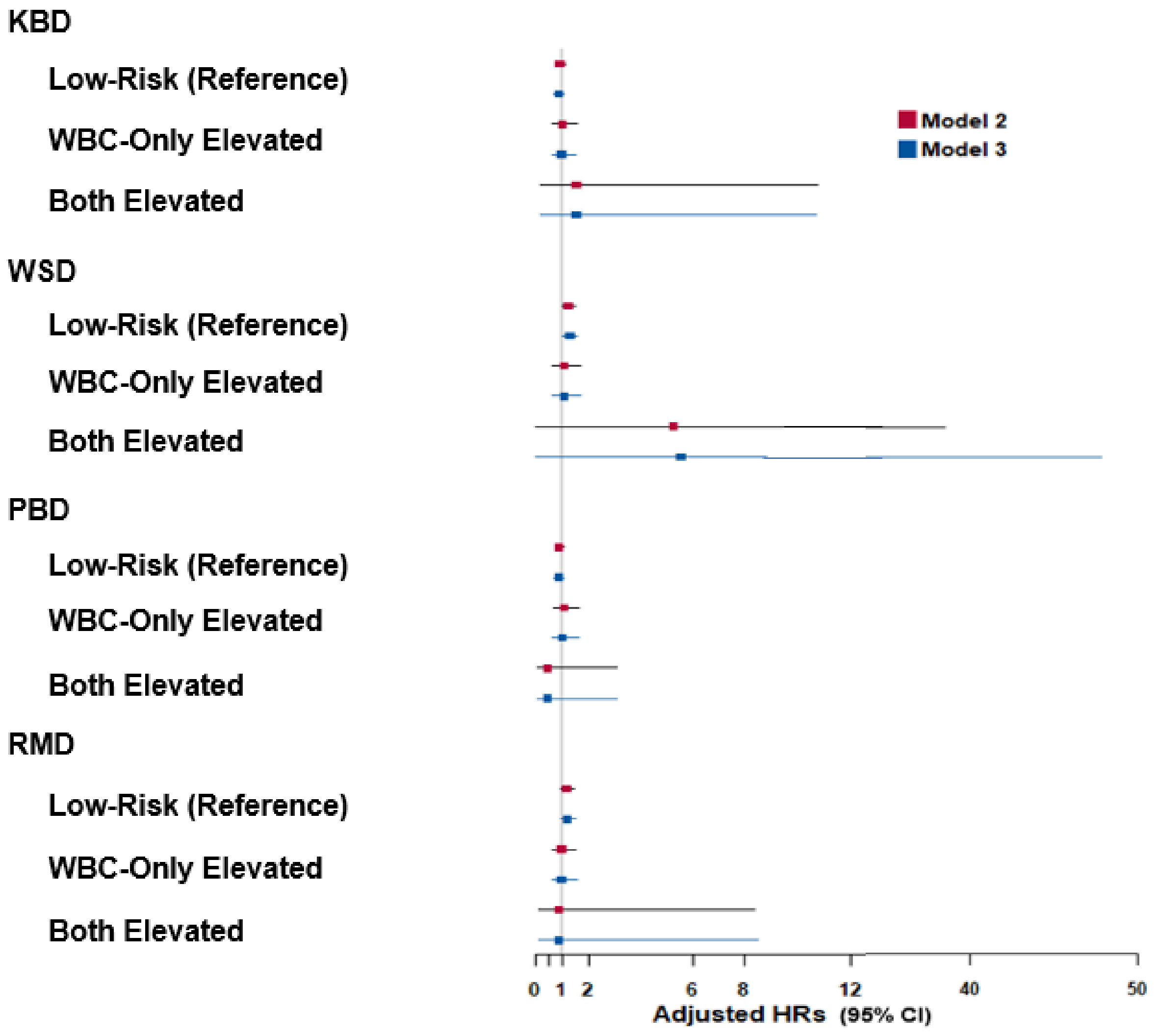
KBD
Low-Risk (Reference)
WBC-Only Elevated
Both Elevated
WSD
Low-Risk (Reference)
WBC-Only Elevated
Both Elevated
PBD
Low-Risk (Reference)
WBC-Only Elevated
Both Elevated
RMD
Low-Risk (Reference)
WBC-Only Elevated
Both Elevated
Model 2
Model 3
0
1
2
6
8
12
40
50
Adjusted HRs (95% CI)

Figure S6B

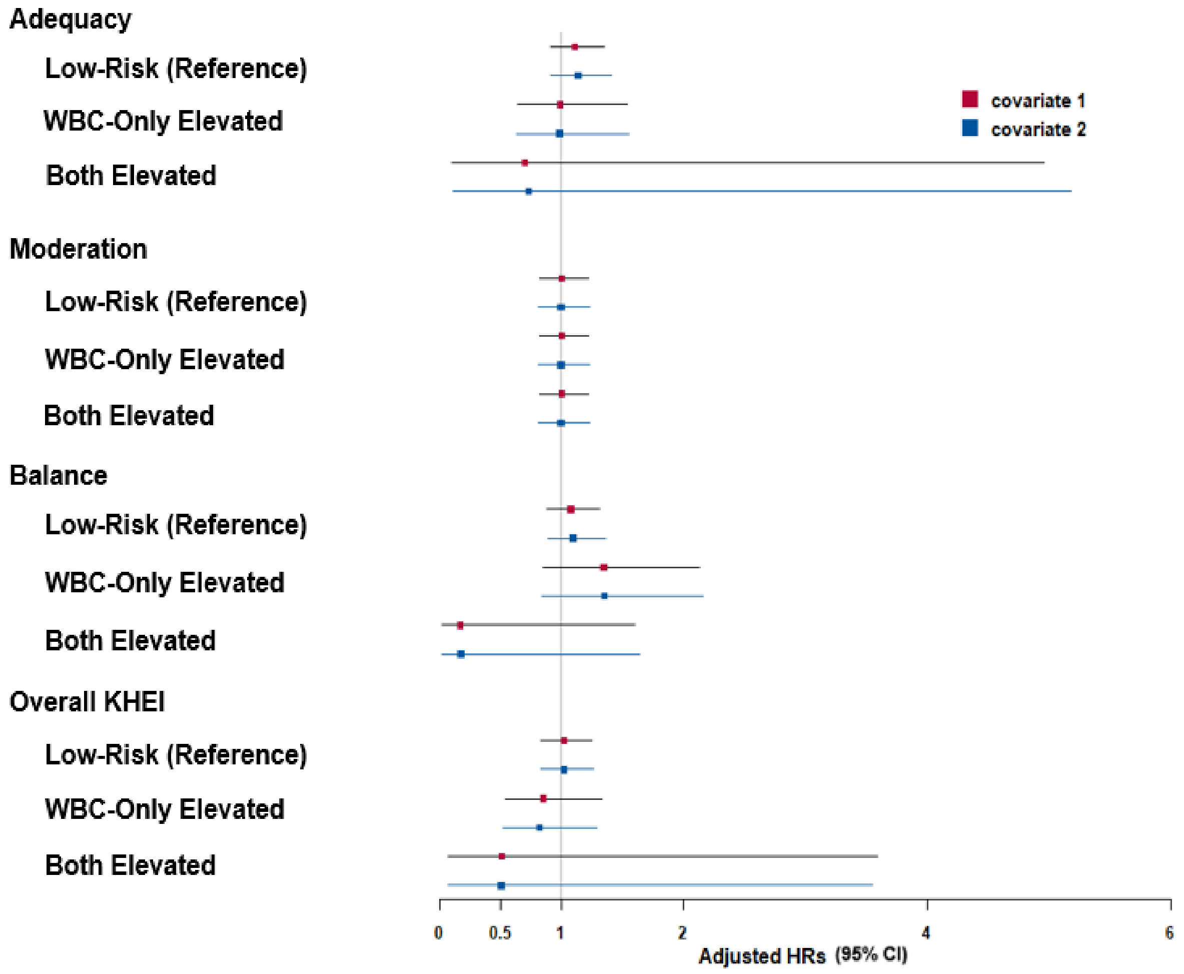
Adequacy
Low-Risk (Reference)
WBC-Only Elevated
Both Elevated
Moderation
Low-Risk (Reference)
WBC-Only Elevated
Both Elevated
Balance
Low-Risk (Reference)
WBC-Only Elevated
Both Elevated
Overall KHEI
Low-Risk (Reference)
WBC-Only Elevated
Both Elevated
covariate 1
covariate 2
0
0.5
1
2
4
6
Adjusted HRs (95% CI)

Figure S6C

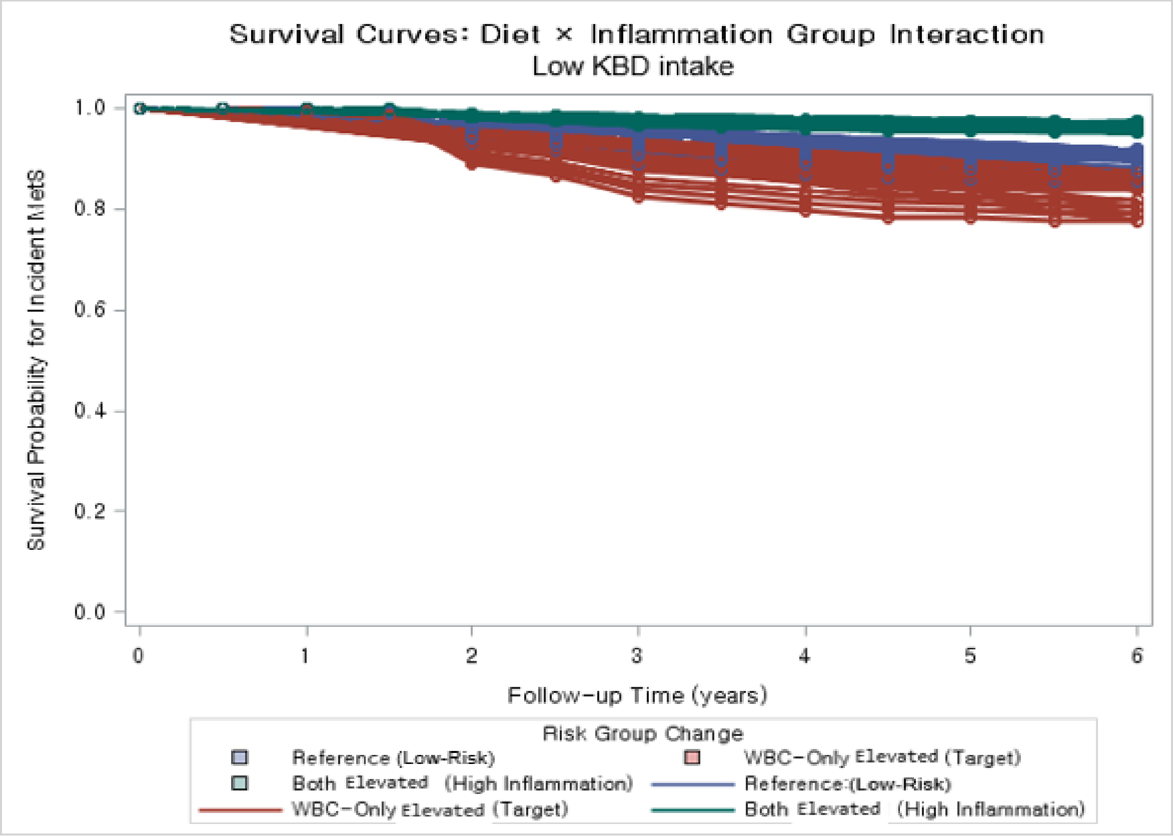
Survival Curves: Diet × Inflammation Group Interaction
Low KBD intake
Survival Probability for Incident MetS
1.0
0.8
0.6
0.4
0.2
0.0
0
1
2
3
4
5
6
Follow-up Time (years)
Risk Group Change
Reference (Low-Risk)
WBC-Only Elevated (Target)
Both Elevated (High Inflammation)
Reference (Low-Risk)
WBC-Only Elevated (Target)
Both Elevated (High Inflammation)

Supplementary Tables

Table S1. Adjusted means of nutrient intake, healthy eating index (HEI), and dietary patterns in the 1st recruitment

	Low-Risk (N-WBC/N-CRP) (n=21,828)	Acute inflammation (N-WBC/H-CRP) (n=154)	WBC-Only Elevated (H-WBC/N-CRP) (n=32,187)	Both Elevated (H-WBC/H-CRP) (n=575)
Energy (EER %)	97.1±0.22	97.7±1.99	97.5±0.45	97±2.5
CHO (En%)	71.7±0.05	70.9±0.45	71.4±0.1	71.2±0.56
Protein (g/kg bw)	0.461±0.001^{a}	0.467±0.01^{a}	0.454±0.001^{b}	0.457±0.001^{a*}
Fat (En%)	13.9±0.04	14.4±0.35	14.1±0.08	14.3±0.43
Dietary inflammation index	-20.1±0.12	-19.3±1.04	-20.5±0.24	-19.9±1.31
Flavonoids (mg/d)	40.6±0.24^{a}	40±2.13^{ab}	39.1±0.48^{b}	35.5±2.67^{ab***}
V-C (mg/d)	110±0.39^{a}	108±3.78^{ab}	108±0.76^{b}	102±4.746^{ab***}
V-E (mg/d)	7.89±0.04^{a}	7.58±0.38^{ab}	7.16±0.08^{b}	7.32±0.45^{ab***}
V-D (μg/d)	6.48±0.03^{a}	6.42±0.27^{ab}	6.18±0.06^{b}	6.53±0.32^{ab***}
Fiber (g/d)	14.6±0.05^{a}	14.6±0.44^{ab}	14±0.1^{b}	13.9±0.52^{ab***}
Calcium (mg/d)	443±1.35^{a}	441±12.25^{ab}	435±2.7^{b}	428±14.58^{ab*}
Na (g/day)	2.42±0.01	2.41±0.07	2.37±0.01	2.34±0.08
Coffee (g/day)	3.43±0.02^{b}	3.38±0.19^{b}	3.84±0.04^{a}	3.42±0.24^{b***}
Glycemic index	38.9±0.17	38.9±1.53	36.4±0.34	34.3±1.82
KBD (N, %)	7028 (32.2)	46 (30.1)	10621(33.0)	173 (30.0)
WSD (N, %)	6723 (30.8)	50 (32.2)	12199 (37.9)	221 (38.5)***
PBD (N, %)	7264 (33.3)	47 (30.7)	9386(29.2)	163 (28.4)***
RMD (N, %)	7138 (32.7)	50 (32.4)	9965 (31.0)	214 (37.2)***
Adequacy scores in HEI	34.1±0.05^{a}	33.5±0.42^{ab}	33.7±0.09^{b}	33.4±0.5^{ab***}
Moderation scores in HEI	43.1±0.04	43.1±0.4	42.8±0.09	42.9±0.48
Balance scores in HEI	106±0.36^{a}	103±3.22^{ab}	101±0.71^{b}	96±3.83^{ab***}
Overall HEI scores	90.7±0.06^{a}	89.8±0.55^{ab}	89.7±0.12^{b}	89.5±0.65^{ab***}

Alcohol intake (g/day)	15.8±0.23	15.6±2.04	15.4±0.44	15.6±2.39
Physical activity (min/day)	26.9±0.15[a]	26.2±1.36[ab]	25.9±0.31[b]	25.2±1.71[ab*]
Non-Smoke (N, %)	16,954 (77.7)	117 (76.0)	22,759 (70.7)	367 (63.8)[***]
Former smoke	3,388 (15.5)	29 (18.6)	4,992 (15.5)	111 (19.4)
Smoke	1369 (6.3)	8 (4.9)	4,145 (12.9)	93 (16.2)

Adjusted means ± standard errors or numbers (percentages) are presented. Values were adjusted for age, residential region, education, income, energy intake, drinking status, smoking status, and physical activity. Immune phenotypes were categorized into four groups based on white blood cell (WBC) count (cutoff: 6.2 × 10^9/L) and C-reactive protein (CRP) level (cutoff: 1 mg/dL). Abbreviations: N-, normal; H-, high; EER%, percentage of estimated energy requirement (EER); En%, percentages of daily energy intake; CHO, carbohydrate; KBD, Korean-style balanced diet; WSD, Western-style diet; PBD, plant-based diet; RMD, rice-main diet; HEI, healthy eating index. [*]$P < 0.05$ and [***]$P < 0.001$ indicate significant differences among immune phenotypes. Different superscript letters (a, b, c, d) denote significant pairwise differences according to Tukey’s test ($P < 0.05$).

Table S2. Genome-wide significant variants associated with high WBC and normal CRP compared with normal WBC and normal CRP in KoGES (logistic GWAS; $P < 5 \times 10^{-8}$; LD $r^2 < 0.001$)

CHR	SNP	BP	A1	OR	SE	L95	U95	P	Gene names	Location
8	rs1982094	1.31E+08	T	1.135	0.023	1.085	1.187	4.07E-08	*CCDC26*	intron
7	rs445	92408370	T	1.176	0.01978	1.131	1.222	2.78E-16	*CDK6*	intron
7	rs2299242	92428239	G	0.8938	0.01847	0.8621	0.9268	1.23E-09	*CDK6*	intron
7	rs41348	28716848	G	0.9007	0.0189	0.868	0.9347	3.17E-08	*CREB5*	intron
17	rs25645	38173143	A	1.198	0.01887	1.154	1.243	1.06E-21	*CSF3*	3' UTR
2	rs1491165775	1.37E+08	C	0.8184	0.02343	0.7817	0.8569	1.21E-17	*DARS1*	3' UTR
2	rs660002	1.37E+08	T	0.8901	0.01908	0.8574	0.924	1.04E-09	*DARS1*	intron
2	rs2278682	1.37E+08	C	0.8899	0.0191	0.8572	0.9239	1.03E-09	*DARS1, DARS1-AS1*	5' UTR
17	rs56030650	38131187	A	0.8499	0.0184	0.8198	0.8811	9.62E-19	*GSDMA*	missense
6	rs201150636	31170528	G	1.109	0.01837	1.07	1.15	1.74E-08	*HCG27*	Non-coding exon
6	rs9295963	31169494	T	0.9036	0.01851	0.8714	0.9369	4.26E-08	*HCG27*	intron
6	rs1050328	31238155	G	0.8688	0.01911	0.8369	0.902	1.83E-13	*HLA-C*	missense
6	rs796393201	31236687	A	0.8778	0.02221	0.8404	0.9169	4.39E-09	*HLA-C*	3' UTR
6	rs1050428	31239543	T	0.8439	0.02902	0.7973	0.8933	5.03E-09	*HLA-C*	missense
6	rs1050373	31239016	A	0.845	0.02904	0.7982	0.8945	6.65E-09	*HLA-C*	missense
6	rs1050437	31239585	T	1.112	0.01912	1.071	1.154	2.83E-08	*HLA-C*	missense
6	rs10093	32609173	C	1.113	0.01845	1.074	1.154	5.77E-09	*HLA-DQA1*	missense
6	rs28366215	32557310	C	0.8922	0.02047	0.8572	0.9288	2.55E-08	*HLA-DRB1*	Nmd transcript

6	rs199516615	32493510	A	0.9018	0.01887	0.8691	0.9358	4.27E-08	*HLA-DRB5*	intron
2	rs3754689	1.37E+08	T	0.8993	0.01918	0.8661	0.9337	3.12E-08	*LCT*	missense
2	rs4988285	1.37E+08	A	0.838	0.02366	0.8	0.8777	7.86E-14	*MCM6*	3’ UTR
17	rs2302777	38179492	G	1.201	0.01889	1.157	1.246	3.39E-22	*MED24*	3’ UTR
6	rs28360042	31001781	T	1.111	0.01876	1.071	1.153	1.84E-08	*MUC22*	intron
6	rs887464	31145920	T	1.112	0.01869	1.072	1.154	1.35E-08	*POU5F1*	intron
17	rs7021	38153875	A	0.8405	0.01841	0.8107	0.8713	3.67E-21	*PSMD3*	3’ UTR
2	rs74459131	1.36E+08	T	0.8393	0.02381	0.801	0.8794	1.84E-13	*R3HDM1*	intron
17	rs62065216	38218773	G	1.129	0.01843	1.089	1.171	4.29E-11	*THRA*	5’ UTR
2	rs76260229	1.37E+08	T	0.8402	0.02362	0.8022	0.8801	1.73E-13	*UBXN4*	3’ UTR
2	rs186775077	1.37E+08	T	0.8495	0.02443	0.8098	0.8912	2.49E-11	*UBXN4*	Nmd transcript
6	rs2524070	31244520	A	0.8415	0.02829	0.7961	0.8895	1.07E-09	*USP8P1*	Non-coding exon
2	rs16831432	1.36E+08	C	1.111	0.0185	1.071	1.152	1.26E-08	*ZRANB3*	Nmd transcript

Genome-wide significant variants are shown at $P < 5 \times 10^{-8}$. Models were adjusted for age, residential region, education, income, energy intake, drinking status, smoking status, and physical activity. The table includes chromosome (CHR), single nucleotide polymorphism (SNP), base-pair position (BP), minor allele (A1), odds ratio (OR), standard error (SE), P-value, nearest gene (gene names), genomic location (location).

Table S3. Linear associations of genome-wide significant variants identified by logistic GWAS with the high WBC and normal CRP phenotype in KoGES and UK Biobank

				KoGES						UK Biobank					
CHR	SNP	BP	Risk allele	A1	REF	Beta	Beta STD	SE	P	A1	REF	Beta	Beta STD	SE	P
2	rs1260326	27730940	C	C	T	-0.088	-0.088	0.011	5.60E-16	T	C	0.038	-0.038	0.005	1.75E-14
2	rs76260229	136511617	G	T	G	0.139	-0.139	0.014	3.11E-23	T	G	0.146	-0.146	0.065	0.0251
2	rs3754689	136590746	C	T	C	0.08	-0.08	0.011	1.10E-12	T	C	-0.014	0.014	0.007	1.54E-08
2	rs309133	136751842	A	C	A	0.088	-0.088	0.011	3.62E-15	C	A	0.009	-0.009	0.007	0.212
6	rs887464	31145920	T	T	C	-0.072	-0.0715	0.011	4.62E-11	T	C	-0.084	-0.084	0.005	4.82E-66
6	rs1050428	31239543	T	T	C	0.12	0.12	0.011	1.16E-26	T	C	0.131	0.013	0.006	1.76E-79
6	rs10093	32609173	C	C	G	-0.079	-0.079	0.011	1.55E-13	G	C	0.1	-0.1	0.005	1.71E-78
7	rs41348	28716848	A	G	A	0.068	-0.068	0.011	6.71E-10	G	A	0.057	-0.057	0.005	2.51E-30
7	rs445	92408370	T	T	C	-0.134	-0.134	0.011	1.26E-31	T	C	-0.148	-0.148	0.008	4.40E-75
17	rs56030650	38131187	C	A	C	0.128	-0.128	0.011	1.25E-32	A	C	0.143	-0.143	0.005	2.81E-189
17	rs7021	38153875	T	A	T	0.139	-0.139	0.011	6.42E-38	A	T	0.158	-0.158	0.005	2.65E-218
17	rs25645	38173143	A	A	G	-0.142	-0.142	0.011	7.93E-39	A	G	-0.113	-0.113	0.005	3.36E-110
17	rs2302777	38179492	G	G	A	-0.144	-0.144	0.011	7.40E-40	G	A	-0.112	-0.112	0.005	9.36E-108

Genome-wide significant variants are shown at $P < 5 \times 10^{-8}$. Models were adjusted for age, residential region, education, income, energy

intake, drinking status, smoking status, and physical activity. The table includes chromosome (CHR), single nucleotide polymorphism (SNP), base- pair position (BP), minor allele (A1), major allele (A2), risk allele (Risk), odds ratio (OR), standard error (SE), P-value, nearest gene (gene names), genomic location (location), minor allele frequency (MAF), and Hardy–Weinberg equilibrium (HWE).
